# Mesenchymal stem cells and the central nervous system: historical perspectives and future directions

**DOI:** 10.3389/fnmol.2026.1742864

**Published:** 2026-02-23

**Authors:** Christopher Y. Mazurek, Julia K. Kaniuk, Christopher S. Ahuja

**Affiliations:** 1 Feinberg School of Medicine, Northwestern University, Chicago, IL, United States; 2 Department of Neurological Surgery, Northwestern Medicine, Chicago, IL, United States; 3 Simpson-Querrey Research Institute, Chicago, IL, United States

**Keywords:** central nervous system, clinical trials, immunomodulation, inflammation, mesenchymal stem cell, MSC secretome, neurodegeneration, regenerative medicine

## Abstract

Mesenchymal stem cells (MSCs) have been studied as a potential therapy for a wide range of conditions for approximately 30 years. MSCs have shown promise in treating pathologies of or affecting the central nervous system (CNS), specifically Alzheimer’s disease (AD), Parkinson’s disease (PD), amyotrophic lateral sclerosis (ALS), multiple sclerosis (MS), stroke, spinal cord injury (SCI), traumatic brain injury (TBI), degenerative disc disease (DDD), and sepsis/meningitis. The therapeutic benefits of MSCs derive primarily from their arsenal of secreted factors that promote anti-inflammatory and pro-survival pathways while attenuating harmful immune responses, thus making them powerful immunomodulatory entities which are also capable of affecting a diverse range of cellular functions to promote endogenous mechanisms of repair. This review summarizes the current state of clinical trials research regarding pathologies of the CNS with a focus on historical progression and upcoming trials. We take a mechanistic approach to explain the therapeutic basis of MSCs and how this has informed clinical trials. We also mention the role of the MSC secretome and MSC exosomes in the treatment of CNS pathologies as well as their increasing use in clinical trials. Finally, we address the challenges inherent to the clinical translation and implementation of MSC therapies along with future directions of the field.

## 1 Introduction and history

Stem cells are cells that are capable of self-renewal and differentiation into other cell types. Because of this unique ability, they carry great potential for enhancing the regeneration of damaged tissue and the growth of new tissue (McCulloch and Till, 2005). Common sources of stem cells include embryologic stem cells, fetal stem cells, induced pluripotent stem cells (iPSCs), and mesenchymal stem cells (MSCs) (Bacakova et al., 2018). Stem cells obtained from human embryos are either totipotent or pluripotent depending on when they were collected and are therefore desirable for research and clinical uses. However, there is significant ethical concern surrounding the use of embryologic stem cells (Wertz, 2002). iPSCs offer another source of pluripotent stem cells. They are created through selective expression of specific transcription factors which transform terminally differentiated adult cells back into stem cells (Takahashi et al., 2007; Yu et al., 2007). A major drawback of both iPSCs and embryonic stem cells is their tumorigenicity (Ben-David and Benvenisty, 2011).

MSCs, on the other hand, are promising agents for regenerative therapies due to their convenient methods of procurement, relative lack of tumorigenicity and immunogenicity, and lack of ethical issues that plague embryonic stem cells (Bacakova et al., 2018). Many MSC therapies also benefit from ease of regulation as minimally manipulated cell products (Marks and Gottlieb, 2018). MSCs are a type of multipotent stem cell capable of differentiating into bone, adipose tissue, cartilage, tendon, muscle, and marrow stroma (Pittenger et al., 1999), and have been historically cultured from bone marrow (Pittenger et al., 2019), adipose tissue (Zuk et al., 2001), umbilical cord tissue (Romanov et al., 2003), and placental tissue (In ’t Anker et al., 2004).

**FIGURE 1:**
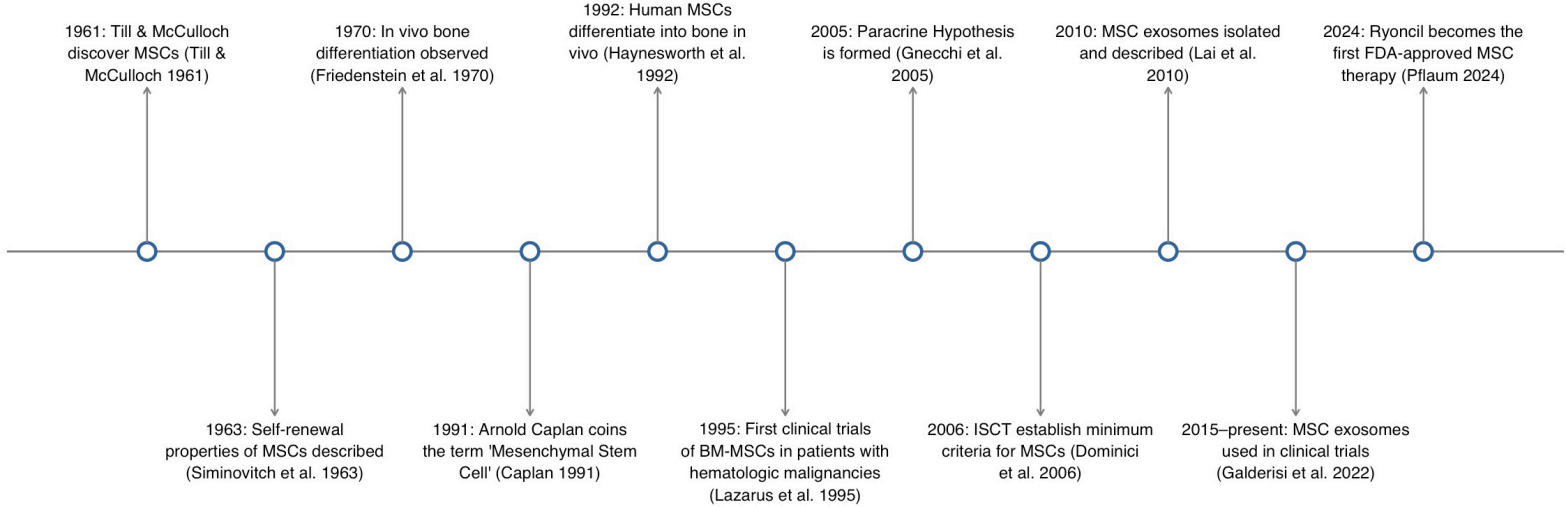
Illustrates a timeline of important events in the development MSC research.

MSCs were first discovered by James Till and Earnest McColloch in 1961. They observed that populations of bone marrow cells injected into irradiated mice resulted in colony formation on the spleens of injected mice, and that the number of observed colonies correlated very closely to the number of injected marrow cells. Till and McCulloch theorized that the colonies may be clonal in nature, derived from “a very small number of cells and possibly even one cell” (Till and McCulloch, 1961). They would prove the clonal nature of these colonies in a follow-up experiment in which marrow cells were irradiated to induce abnormal karyotypes and then injected into irradiated mice. The vast majority of cells from each arising spleen colony carried unique karyotypes, providing overwhelming evidence for the clonal nature of the spleen colonies and proving the differentiation and proliferation potential of cells within bone marrow (Becker et al., 1963). The same research group would go on to describe the self-renewal properties of these mouse spleen colonies, and in doing so described the first theory of bone marrow hematopoietic stem cells (Siminovitch et al., 1963). The ability of marrow cells to differentiate into other tissue lines, like bone, *in vivo* was reported in 1970 by Friedenstein et al. (1970) and replicated in 1989 (Ohgushi et al., 1989). In 1991, Arnold Caplan described the multi-lineage potential of these cells and famously coined the term “mesenchymal stem cells” (Caplan, 1991). In the years that followed, human bone marrow mesenchymal stem cells (BM-MSCs) were transplanted into mice and shown to differentiate into human bone, providing the first evidence for the *in vivo* bone forming capacity of human MSCs (Haynesworth et al., 1992). The first in-human clinical trials would be performed 3 years later, in which autologous BM-MSCs were administered intravenously to patients with hematologic malignancies in remission (Lazarus et al., 1995). No adverse reactions to the treatment were observed, demonstrating for the first time the feasibility and therapeutic potential of MSCs for human disease (Lazarus et al., 1995). Many clinical trials on MSCs have been conducted since this first experiment, but the first and only U.S. Food and Drug Administration (FDA)-approved MSC therapy is Ryoncil (Remestemcel-L-rknd), approved on Dec. 18th, 2024, for the treatment of steroid-refractory graft-vs-host disease in pediatric patients older than 2 months (Kurtzberg et al., 2020; Pflaum, 2024). Figure 1 provides a brief overview of important events in the history of MSC research.

As the field evolves, the therapeutic uses of MSCs have expanded drastically. This review will focus on our current understanding of MSCs and their prospective applications in treating pathologies of the CNS as well as future directions for therapy.

## 2 MSC characterization and sourcing

In 2006, the International Society for Cellular Therapy published minimum guidelines for the characterization of MSCs to enable consistency in research and clinical applications (Dominici et al., 2006). These guidelines include the presence of the specific surface antigens CD73, CD90, and CD105, and the absence of CD34, CD45, CD14 or CD11b, CD79a or CD19, and HLA-DR. Additionally, the stem cells must demonstrate capacity for trilineage differentiation, including osteoblasts, chondrocytes, and adipocytes, which is confirmed through *in vitro* differentiation assays (Wilson et al., 2021). Lastly, the stem cells must be plastic-adherent under standard conditions (Dominici et al., 2006; Fernández Vallone et al., 2013).

MSC therapies are further classified by whether they meet criteria for minimally manipulated cell products or more than minimally manipulated cell products. Minimal manipulation is defined by the FDA as “for structural tissue, processing that does not alter the original relevant characteristics of the tissue relating to the tissue’s utility for reconstruction, repair, or replacement, and for cells or non-structural tissues, processing that does not alter the relevant biological characteristics of cells or tissues”(U.S. Food and Drug Administration, 2025). While most MSC products can be considered minimally manipulated, there are a growing number of new MSC technologies that fall under Section 351 of the Public Health and Safety Act which includes “cell and tissue products that are cultured or more than minimally manipulated, not intended for homologous use, combined with a drug or device, or are allogenic” (Murphy et al., 2013; United States, 2025). This distinction is important because it affects whether manufacturers must submit a biologics license application with the FDA and obtain premarket approval (Marks and Gottlieb, 2018).

There are many different tissue sources of MSCs which are used in preclinical and clinical studies. Human umbilical cord-derived MSCs (hUC-MSC) and Wharton’s Jelly-derived MSCs (WJ-MSCs) are both derived from fetal umbilical cord tissue (Romanov et al., 2003), while placenta-derived MSCs are obtained from human placental tissue and even amniotic fluid (In ’t Anker et al., 2004). Wharton’s Jelly is the largest component of the umbilical cord. It is comprised of the thick connective tissue within the umbilical cord itself. WJ-MSCs refer to MSCs recovered from specifically from this layer of tissue. hUC-MSCs is a broader term referring to MSCs retrieved from perivascular regions surrounding the umbilical arteries and veins, and the umbilical cord lining (Subramanian et al., 2015).

The most common adult tissue sources of MSCs include bone marrow and adipose tissue. BM-MSCs are typically sourced from bone marrow aspiration of the iliac crest (Pittenger et al., 2019). Adipose-derived MSCs (AD-MSCs) can be sourced from either subcutaneous or visceral adipose tissue deposits via liposuction or surgical resection (Zuk et al., 2001; Schneider et al., 2017; Bunnell, 2021). Both BM-MSCs and AD-MSCs are typically isolated, cultured, and expanded *in vitro* before administration to the patient (Pittenger et al., 2019). Bone marrow mononuclear cells (BMMNCS), on the other hand, are not extensively processed prior to administration. BMMNCs refer to a heterogenous mixture of cell types (MSCs, endothelial stem cells, hematopoietic stem cells, and lymphocytes) obtained from bone marrow that can be used in a regenerative capacity. BMMNCs as a formulation do not undergo extensive *in vitro* isolation or culturing prior to administration (Du et al., 2021). Other sources of MSCs exist, such as skin (Melo et al., 2017) and human exfoliated deciduous teeth (Miura et al., 2003), but these are used far less commonly in CNS pathologies.

MSCs are found in different concentrations depending on which tissue they are collected from, and estimates can vary considerably. For example, BM-MSCs may contain anywhere from 10 to 83 colony-forming units (CFU) per 10^6^ nucleated cells, whereas MSCs in adipose tissue are much more plentiful with CFU frequency between 20 and 51,000 (Wexler et al., 2003; Murphy et al., 2013; Heo et al., 2016). Additionally, there is conflicting evidence surrounding the CFU frequency of MSCs derived from umbilical cord blood (Kern et al., 2006; Heo et al., 2016). MSCs sourced from different tissues also exhibit specific differentiation profiles and growth rates. BM-MSCs and AD-MSCs share similar proliferation rates (24–48 h) (Riekstina et al., 2009), but BM-MSCs do not demonstrate stable proliferation, entering senescence far sooner than AD-MSCs or hUC-MSCs (Kern et al., 2006; Hayashi et al., 2008). In contrast, MSCs derived from dental tissue appear to proliferate much faster and can sustain higher passage numbers compared to BM-MSCs and AD-MSCs (Chamila Prageeth Pandula et al., 2014; Zhang et al., 2018). Significant differences in CFU frequency and proliferation rates may be due to inter-individual difference in MSC viability.

MSCs also differ by method of collection. A significant disadvantage of BM-MSCs is that they require aspiration of the bone marrow, a painful and invasive procedure often requiring general anesthesia and hospitalization (Gendron et al., 2019; Murakami et al., 2024). AD-MSCs, on the other hand, are plentiful within adipose tissue and easily accessible through minimally invasive lipoaspiration (Mazini et al., 2019). hUC-MSCs and WJ-MSCs have an advantage in that they can be procured non-invasively after birth (Beeravolu et al., 2017). While trilineage differentiation into bone, cartilage, and adipose tissue is common to all MSCs, they do not share equal proclivity for all tissue types. BM-MSCs have the highest osteogenic differentiation capacity (Xu et al., 2017). AD-MSCs of course show greatest capacity for adipose tissue differentiation, but lesser capacity for chondrocyte differentiation compared to BM-MSCs (Mohamed-Ahmed et al., 2018). hUC-MSCs show moderate ability to differentiate into bone, cartilage, and adipose tissue (Nagamura-Inoue and He, 2014), but appear to have the highest potential for osteogenic differentiation (Baksh et al., 2007). WJ-MSCs, when compared to hUC-MSCs, showed greater osteogenic and chondrocyte differentiation capacity and were easier to harvest and culture (Subramanian et al., 2015). Beyond these three tissue types, MSCs from various sources have also been shown to differentiate into neural tissue, cardiomyocytes, and hepatocytes under the right conditions (Rebelatto et al., 2008).

### 2.1 Mesenchymal stem cell mechanisms of action

**FIGURE 2:**
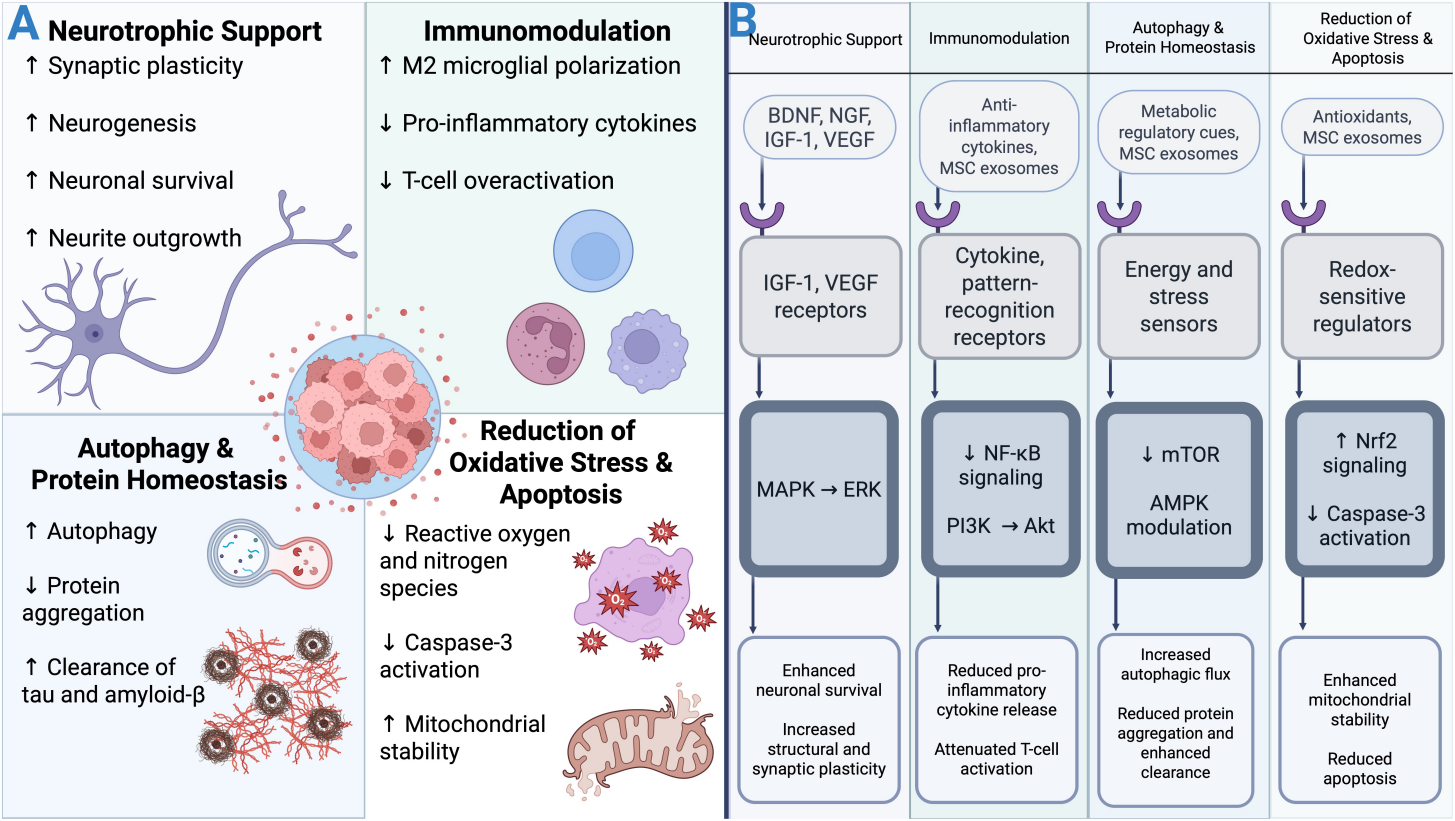
**(A)** General schema of MSC mechanisms of action. **(B)** Mechanistic signaling pathways mediating MSC secretome–driven neuroprotection and immunomodulation. MSC-derived soluble factors and exosomes engage target cell receptors and sensing machinery to activate convergent intracellular signaling hubs across four functional domains. Neurotrophic signaling via IGF-1 and VEGF receptors activates the MAPK–ERK pathway, promoting neuronal survival and structural plasticity. Immunomodulatory cues suppress NF-κB signaling while promoting the PI3K-Akt pathway, resulting in reduced pro-inflammatory cytokine release and attenuation of immune cell activation. Regulation of autophagy and protein homeostasis is mediated through increased AMPK signaling and reduced mTOR signaling, facilitating increased autophagic flux and clearance of aggregated proteins. Oxidative stress reduction and cell survival are supported through Nrf2 activation and inhibition of caspase-3–dependent apoptosis, preserving mitochondrial stability. Collectively, these signaling pathways provide a mechanistic framework linking MSC secretome activity to the functional outcomes summarized in **(A)**. Created in BioRender. Ahuja, C. (2026) (https://BioRender.com/nuc61cy).

MSCs exert a diverse range of neuroprotective and regenerative functions through multifaceted interactions with neural and immune cell populations, as well as through their robust secretory activity. It was originally postulated that the therapeutic potential of stem cells was due to direct cellular engraftment into wounded or diseased tissue; however, it was found that only about 5% of MSCs actually integrate in the host tissue (Pittenger et al., 2019). A landmark study by Gnecchi et al. articulated for the first time that the therapeutic activity of MSCs is predominantly mediated through paracrine mechanisms rather than direct differentiation into local cell subtypes (Gnecchi et al., 2005). In 2010, Lai et al. confirmed that exosomes released from MSCs were the mediators of this observed paracrine activity (Lai et al., 2010). While MSCs can migrate to sites of injury in the CNS, only a minority undergo neural lineage commitment, underscoring the primacy of their secretome in driving neuroprotective, immunomodulatory, and regenerative effects. An overview of the mechanisms of action of MSCs are illustrated in Figure 2.

### 2.2 Modulating the immune response

MSCs and their secretomes promote a shift toward an anti-inflammatory M2 microglial phenotype in multiple murine models of neurological disease, while simultaneously inhibiting neurotoxic astrocyte activation. This dual action reduces glutamate excitotoxicity and restores neurotransmitter balance, particularly relevant in the context of AD (Colonna and Butovsky, 2017; Chai et al., 2024; Saglam-Metiner et al., 2024). For instance, Liu et al. demonstrated that BM-MSC exosome administration in AD mouse models improved behavioral outcomes and reduced astrocytic activation, leading to diminished amyloid-beta (Aβ) and tau burden (Liu et al., 2022). The MSC secretome also inhibits neuronal apoptosis *in vitro* and *in vivo*, elevates dopamine levels, and enhances microglial autophagy (Chen H. X. et al., 2020). Moreover, MSCs limit glial scar formation, thereby fostering a more permissive environment for neural repair (Anjum et al., 2020; Yang et al., 2021).

Promotion of autophagy is another critical MSC function, with implications across neurodegenerative and systemic inflammatory disorders. BM-MSCs induce microglial autophagy, facilitating clearance of Aβ and tau in AD and a-synuclein aggregates in PD, potentially through Beclin-1–dependent mechanisms (Park et al., 2014; Shin et al., 2014; Chen H. X. et al., 2020). In sepsis, MSC-mediated modulation of immune cells enhances autophagic processes, thereby mitigating inflammation and reducing the risk of multiorgan failure (Krasnodembskaya et al., 2010; Mei et al., 2010; Jackson et al., 2016; Laroye et al., 2017; Morrison et al., 2017). This may involve direct mitochondrial transfer from MSCs to host macrophages, augmenting their capacity for autophagic clearance (Jackson et al., 2016; Morrison et al., 2017). This increased autophagic activity is associated with downregulation of the mTOR pathway, a major regulator of cell metabolism and protein homeostasis (Querfurth and Lee, 2021). Indeed, inhibition of mTOR signaling via inhibitors like rapamycin leads to an increase in autophagic activity and reduction in neurotoxic protein aggregations in certain neurodegenerative (Crino, 2016; Querfurth and Lee, 2021). Multiple *in vivo* and *in vitro* studies have shown that autophagy induced by MSCs is associated with reduced mTOR signaling and increased AMPK, a negative regulator of mTOR (Kim K. W. et al., 2015; Böttcher et al., 2016; Vasandan et al., 2016; Zheng et al., 2018).

MSCs also modulate peripheral immune responses, limiting T-cell–mediated contributions to neuroinflammation (Krasnodembskaya et al., 2010; Mei et al., 2010; Zhang et al., 2013; Laroye et al., 2017). In AD trials, MSCs inhibit T-cell proliferation and reduce infiltration into tau-rich brain regions, likely via secretion of immunoregulatory mediators such as TGF-β1, hepatocyte growth factor, prostaglandin E2, indoleamine 2,3-dioxygenase, and nitric oxide (NO) (Pang et al., 2021). Experimental models of viral encephalitis and TBI have shown that MSCs downregulate inflammatory microglial activation, preserve blood brain barrier (BBB) integrity, suppress peripheral immune cell invasion, and promote neuronal survival (Bian et al., 2017).

Through their secretory profile, MSCs induce the release of anti-inflammatory cytokines and neurotrophic factors. They downregulate pro-inflammatory mediators (TNF-α, IFN-γ, IL-1β) while upregulating IL-10 and IL-1 receptor antagonist (IL-1RA) (Yang et al., 2020). MSC-derived factors such as VEGF, BDNF, IGF-1, GDNF, NGF, FGF2, NT4/5, and PDGF enhance synaptogenesis, neurogenesis, oligodendrocyte survival, and remyelination (Kang et al., 2003; Jaramillo-Merchán et al., 2013; Marconi et al., 2013; Glenn et al., 2015). Such trophic support underlies observed improvements in cognition and memory in preclinical AD models, as well as remyelination in MS and enhanced neuronal survival in ALS models (Xie et al., 2016; Lim et al., 2020; Park et al., 2021).

### 2.3 Promotion of tissue survival

An additional dimension of MSC action is their anti-apoptotic effect. By inhibiting caspase activation, particularly caspase 3, and upregulating anti-apoptotic proteins such as survivin and seladin-1, MSCs promote cell survival. Engineered MSCs overexpressing Bcl-2 further enhance tissue regeneration (Hyun et al., 2013; Qin et al., 2022). In TBI models, MSC treatment increases neurotrophin levels while reducing apoptotic signaling, correlating with significant functional recovery (Kim H. J. et al., 2010).

Promotion of neurogenesis constitutes another important MSC-mediated process. Studies have demonstrated that MSCs stimulate subventricular zone neurogenesis, bridge lesion gaps via axonal guidance structures, and activate endogenous repair mechanisms in the spinal cord. These effects extend to promoting oligodendrocyte maturation and remyelination, thereby restoring white matter integrity (Kan et al., 2011; Jeong et al., 2014; El-Akabawy and Rashed, 2015).

MSCs exert neuroprotective and neuro-regenerative effects largely through the secretion of neurotrophic factors, such as BDNF and VEGF, and the activation of key intracellular signaling pathways, including MAPK/ERK and PI3K/Akt. MSC-derived cytokines, notably IGF-1, VEGF, and Periostin 2, activate the PI3K/Akt pathway, leading to upregulation of inhibitors of apoptosis proteins and suppression of caspase-3, thereby reducing neuronal apoptosis and promoting neuroprotection (Lu et al., 2025). Furthermore, the PI3K/Akt pathway has been shown to support immunomodulation and promotion of autophagy, MSC exosomes, discussed in more detail in later sections, can activate the MAPK/ERK pathway, which is involved in neuronal differentiation, neurogenesis, and synaptic plasticity (Tzeng et al., 2015; Zhou et al., 2021).

Reduction of oxidative stress further contributes to MSC-mediated neuroprotection (Chen et al., 2001). By decreasing reactive oxygen species (ROS) and associated damage, MSCs and their exosomes counteract oxidative injury in AD, PD, and infectious CNS conditions, where reductions in gliosis and inflammatory cytokine release have also been observed.

### 2.4 Mesenchymal stem cell exosomes

An important contributor to the therapeutic efficacy of MSCs are MSC exosomes. Exosomes, first described in the 1960s (Wolf, 1967), are membrane-bound structures ranging from 30–200 nm in diameter (Phinney and Pittenger, 2017; Joo et al., 2020). They can be formed either from early endosomes via luminal budding of the endosomal membrane or from direct budding of the plasma membrane. Thus, they contain proteins, nucleic acids, and enzymes that are unique to their parent cell (Rabinowits et al., 2009; Joo et al., 2020). MSC-derived exosomes contain a wide array of signaling molecules, including miRNAs, mRNAs, cytokines, lipids, and growth factors that work in concert to attenuate inflammation and promote tissue regeneration (Phinney and Pittenger, 2017; Joo et al., 2020). Indeed, collaborative efforts to categorize all recorded molecules found in exosomes across the scientific literature have identified nearly 50,000 distinct proteins, nucleic acids, lipids, enzymes, signaling molecules, and more (Keerthikumar et al., 2016), though only a subset are responsible for the therapeutic effects of MSCs.

MSC exosomes have emerged as a potent mediator of the therapeutic effects traditionally attributed to MSC transplantation, offering a cell-free approach for treatment (Shimojima et al., 2016; Soundara Rajan et al., 2017; Laso-García et al., 2018; Wang H. et al., 2019; Ahmadvand Koohsari et al., 2021; Galderisi et al., 2022). One of the most extensively characterized effects of MSC-derived exosomes is their ability to attenuate neuroinflammation. BM-MSC-derived exosomes administered intrathecally have been shown to reduce microglial and astrocytic activation, lowering inflammatory cytokines such as IL-1β, IL-6, and TNF-α, as well as decreasing Aβ and phosphorylated tau expression (Wang et al., 2018; Kaur et al., 2019; Li et al., 2021a; Gan and Ouyang, 2022; Liu et al., 2022). Liu et al. demonstrated that these exosomes suppressed pro-inflammatory A1 astroglial and M1 microglial differentiation while promoting the neuroprotective A2 astroglial phenotype (Liu et al., 2019). Such immunomodulatory effects extend beyond glial phenotypes; in experimental autoimmune encephalomyelitis (EAE) models, MSC-derived exosomes reduced a broad spectrum of inflammatory mediators including TNF-α, IFN-γ, IL-1β, GM-CSF, IL-17, IL-18, and lowered immune cell infiltration, while elevating anti-inflammatory cytokines such as IL-4, IL-10, and TGF-β (Shimojima et al., 2016; Soundara Rajan et al., 2017; Laso-García et al., 2018; Wang H. et al., 2019; Ahmadvand Koohsari et al., 2021). These effects correlate with improved motor and cognitive function in models of PD, AD, and MS (Wang et al., 2018; Kaur et al., 2019; Kim et al., 2022).

Cell metabolites found in MSCs contribute substantially to their immunomodulatory effects. *In vitro* studies investigating the contents of MSC-derived exosomes revealed increased numbers of lipids such as ceramide, phospholipids, phosphatidylethanolamine, and lysophosphatidylcholines, which facilitate intercellular signaling (Showalter et al., 2019). Many metabolites have also been identified in MSC exosomes, including adenosine, glutamine, arginine, aspartic acid, and 5’-deoxy-5’-methylthioadenosine, among others (Showalter et al., 2019). These metabolites have been shown to modulate the immune response through regulation of macrophages and T-lymphocytes (Yeramian et al., 2006; Hnia et al., 2008; Haskó and Pacher, 2012; Haskó and Cronstein, 2013; Ohta and Sitkovsky, 2014; Henrich et al., 2016; Kim and Kim, 2017; Palmieri et al., 2017).

Beyond their immunomodulatory properties, MSC exosomes exert significant anti-apoptotic effects. Multiple studies have shown their ability to prevent neuronal apoptosis following injury, thereby preserving neuronal integrity in neurodegenerative and ischemic conditions (Li et al., 2021c; Gan and Ouyang, 2022; Liu et al., 2022). In models of AD, MSC-derived extracellular vesicles not only reduced Aβ and tau accumulation but also diminished oxidative stress through suppression of inducible nitric oxide synthase (iNOS) (Wang et al., 2018; Kaur et al., 2019).

In addition to proteins, lipids and other metabolites, MSC-exosomes have been shown to contain a myriad of miRNAs, including mi-133b, miR-223, miR-221, miR-21-5p, miR-181a-2-3p miR-467f, miR-466q, and miR-110a-5p among others (Xin et al., 2013b; Yu et al., 2013; Wang et al., 2015; Reis et al., 2018; Ma et al., 2022; He et al., 2023). Transport of regulatory miRNAs is a key mechanism by which MSC-exosomes modulate inflammation and promote regeneration. For example, miR-181a-2-3p attenuates oxidative damage in PD models via inhibition of EGR1 and NOX4, a driver of the p38/MAPK oxidative stress pathway (Rodríguez et al., 2020; Ma et al., 2022). Similarly, miR-110a-5p targets NOX4, and MSC exosomes can stimulate the Nrf2 pathway, a master regulator of antioxidant gene expression (Hybertson et al., 2011; He et al., 2023). MiR-21-5p, which is highly expressed in MSC-derived exosomes has been shown to downregulate dendritic cell maturation and activity in *in vitro* models (Reis et al., 2018). Additionally, miR-221 transported in MSC-exosomes exert an anti-apoptotic effect on cardiomyocytes exposed to hypoxic conditions (Yu et al., 2013). MiR-223 also confers cardioprotective effects in sepsis (Wang et al., 2015), possibly through inhibition of ICAM-1 on endothelial cells, thus preventing lymphocyte migration (Liu et al., 2021).

MSC exosomes also promote tissue development and homeostasis through regenerative signaling. They have been found to express Wnt family transcripts (Wnt5a, Wnt5b, Wnt7a, Wnt9a) and elevate Wnt protein expression *in vivo*, supporting tissue repair (Li J. et al., 2022). In SCI models, BM-MSC-derived exosomes reduce lesion size, suppress glial scarring, promote angiogenesis, and stimulate axonal regeneration (Liu et al., 2019). Landmark studies by Xin et al., (2013a) and Doeppner et al. demonstrated that BM-MSC exosomes alone could replicate the therapeutic benefits of MSCs in middle cerebral artery occlusion (MCAO) models with agents such as rosuvastatin further reducing inflammasome activation (NLRP1, NLRP3) and astroglial activation (Xin et al., 2013a; Doeppner et al., 2015; Cunningham et al., 2018).

These regenerative effects extend beyond the CNS. In degenerative disc disease (DDD), MSC-derived exosomes inhibit apoptosis of nucleus pulposus (NP) cells, block extracellular matrix degradation, promote matrix reconstruction, attenuate inflammation and oxidative stress, drive chondrocyte differentiation, prevent endplate chondrocyte calcification, and limit annulus fibrosus degeneration (Marbán, 2018; Li et al., 2020; Xie et al., 2020; Zhang J. et al., 2020; Zhang et al., 2021). Similarly, in bone repair, MSC exosomes promote osteogenesis, angiogenesis, chondrogenesis, and immunomodulation, improving outcomes in osteoporosis and fracture healing.

Collectively, MSC-derived exosomes represent a versatile therapeutic platform capable of modulating immune responses, reducing oxidative and apoptotic injury, and promoting tissue regeneration across diverse pathological contexts. Their ability to replicate key MSC-mediated effects without the risks associated with cell transplantation positions them as a promising frontier in regenerative medicine.

## 3 MSCs and CNS pathologies

### 3.1 Alzheimer’s disease

Alzheimer’s Disease is a neurodegenerative disease characterized by progressive memory loss and behavioral changes. Globally, AD is a major contributor to disability-adjusted life years in those over 60 years old (Ballard et al., 2011) and a significant financial burden to families and caregivers (Alzheimer’s Association, 2016). The number of people living with AD in America by 2050 is expected to exceed 13.8 million (Alzheimer’s Association, 2016), and globally this number is estimated to exceed 81 million by 2040 (Ballard et al., 2011), making it a major public health concern. Given its devastating emotional and financial toll on patients and their families, as well as its status as the most common cause of dementia, AD has been the focus of significant therapeutic investment, with MSCs emerging as promising candidates in both preclinical and clinical trials.

The pathological hallmarks of AD are Aβ plaques and neurofibrillary tau tangles in the brain, though recent work has implicated neuroinflammation as a mechanistic driver of AD pathogenesis. Deposition of Aβ plaques is closely associated with pro-inflammatory activation of microglia and astrocytes, resulting in the secretion of IL-1β, IL-6, and TNF-αlpha. These cytokines, in turn, contribute to Aβ plaque formation by propagating the inflammatory microenvironment in the brain (Tuppo and Arias, 2005). Microglia play a key role in clearing cellular debris via autophagy; however, in AD, they shift toward a pro-inflammatory M1 phenotype characterized by reduced autophagic activity and increased cytokine production (Baik et al., 2016; Kaur et al., 2019). Both Aβ plaques and neurofibrillary tau tangles contribute to astrogliosis, with reactive astrocytes commonly found near sites of plaque deposition (Nagele et al., 2003). While initially protective, these astrocytes begin to secrete pro-inflammatory mediators (e.g., TNF-α, IFN-γ, IL-1β, and COX-2) and contribute to glial scar formation (Kaur et al., 2019). Astrocytes have also been implicated in promoting Aβ and tau accumulation through caspase-3 activation (Garwood et al., 2011). In parallel, Aβ plaques induce NO production via the NF-κB pathway, and cytokines such as IFN-γ and TNF-α further elevate NO levels in the AD brain (Akama et al., 1998; Tuppo and Arias, 2005). This creates a self-perpetuating cycle of Aβ deposition and inflammation, leading to high NO and ROS levels, which drive oxidative stress and eventual neuronal apoptosis and necrosis (Kaur et al., 2019).

#### 3.1.1 MSC preclinical trials in AD

Some of the earliest preclinical studies to demonstrate the efficacy of MSCs in treating neurodegenerative disease were performed in Niemann-Pick type C mice (Bae et al., 2005; Bae et al., 2007) with the first AD-specific preclinical study being conducted in the late 2000’s. A study by Lee et al. demonstrated that intracerebral transplantation BM-MSCs into the dentate gyrus reduced Aβ levels and recruited microglia with an autophagy-prone morphology (Lee et al., 2009). In addition to the general MSC mechanisms of action described in previous sections, several studies have shown that human WJ- MSCs and hUC-MSCs not only reduce Aβ load and tau phosphorylation, but also improve memory and cognition in rodent models of AD (Xie et al., 2016; Lim et al., 2020; Park et al., 2021), demonstrating that the changes MSCs make to the biochemical environment of the brain translate to measurable cognitive benefit.

MSC exosomes alone have also been trialed in preclinical models of AD to great effect. Administration of the MSC extracellular vesicles alone mimicked the effects of administration of whole MSCs in mouse models of AD, reducing Aβ plaque levels and markers of microglial and astrocyte activation (Cone et al., 2021; Santamaria et al., 2021). Furthermore, MSC exosomes were shown to transiently increase memory in mice 7 days post administration as measured by the novel object recognition test. However, memory declined again by 14 days post-treatment (Santamaria et al., 2021).

The use of iron oxide nanoparticles (IONP) has also been explored as a method improved targeting of MSCs in murine models of AD. Application of an external magnetic field to the brains of AD mice guided human WJ-MSCs loaded with magnetic dextran-coated IONPs to the hippocampus (Hour et al., 2020). This approach could be further improved when combined with agents or techniques that temporarily disrupt the BBB to enhance MSC entry into the brain (Joshi et al., 2011; Wang et al., 2022). To our knowledge, the use of MSCs augmented with nanoparticles and magnetic fields in conjunction with MSCs has yet to be studied in humans.

#### 3.1.2 MSC clinical trials in AD

Promising pre-clinical results have led to the initiation of many clinical trials investigating the safety and efficacy of MSCs in the treatment of AD; however, most have failed. One reason for this may be because rodent models of AD rely on a familial AD paradigm caused by mutations in *APP*, *PSEN1*, and *PSEN2* (Ballard et al., 2011; Götz et al., 2018; Hernández and García, 2021), whereas in reality, about 95% of AD cases in humans are sporadic (Ballard et al., 2011; Götz et al., 2018). As a result, most preclinical trials, which rely on familial AD models, may be limited in their generalizability for human trials (Drummond and Wisniewski, 2017).

Overall, the published results from completed phase 1 and 2 clinical trials suggest that MSCs are safe and well-tolerated in patients with AD. One of the earliest phase 1 trials to be completed (NCT01297218) found that stereotactic injection of hUCB-MSCs was safe, with no treatment-related adverse events other than pain, headache, and dizziness related to the surgical implantation procedure itself (Kim H. J. et al., 2015). Stereotactic injection is an invasive route of administration and has since been used sparingly in AD trials. While some other trials have attempted to use intraventricular injection (Kim H. J. et al., 2021), most have opted to use intravenous or intranasal routes of administration.

In 2021, Kim et al. published results from another clinical trial (NCT02054208); this time a phase 1/2 double-blind study utilizing the same hUCB-MSCs but at higher doses and with 3 repeat administrations. Consistent with the 2015 study, this trial reported similar safety outcomes. Notably, it also found that MSC injection transiently reduced brain levels of Aβ and tau, although these effects dissipated by 4 weeks (Kim H. J. et al., 2021). A 36-month follow-up study of the same cohort has since been completed (NCT03172117), but to our knowledge the results have not yet been published.

Other clinical trials have investigated MSCs derived from different sources, including adipose tissue. A phase 1/2 study (NCT03117738) tested autologous AD-MSCs in patients with mild-to-moderate AD. MSCs were administered intravenously, and though several serious adverse events were recorded (i.e., diarrhea, acute pulmonary edema, and stage IV esophageal squamous cell carcinoma), the treatment was overall well-tolerated; however, the trial failed to show improvement in measures of cognition, including the ADAS-Cog and the MMSE. A follow-up phase 2b clinical trial of AstroStem cells (NCT04482413) was initiated in 2023 using a similar repeat-dose protocol in a larger study sample, but the trial has passed its estimated completion date.

A phase 1 trial using allogenic BM-MSCs intravenously reported no serious adverse events and noted increases in VEGF and IL-4 levels in treated groups. The IL-4 increase is noteworthy, as IL-4 may regulate apoptosis, microglial activation, and BDNF secretion (Brody et al., 2023). Similarly, a recently published phase 1/2 trial (NCT04388981) investigating allogenic AD-MSC-derived exosomes demonstrated excellent safety and modest cognitive improvement in the medium-dose arm (Xie et al., 2023).

Several upcoming trials are pursuing novel strategies. A phase 1 basket-design study (NCT06607900) is evaluating human umbilical cord MSC-derived exosomes for multiple neurodegenerative diseases, including AD, with completion expected by 2027. A phase 1/2 trial (NCT06775964) in the recruiting phase aims to target the late-presymptomatic/prodromal stage of AD using autologous AD-MSCs. Targeting the early stages of AD may elicit therapeutic benefit before the cascade of neuroinflammation and neurodegeneration becomes irreversible. Another trial will focus specifically on AD patients with behavioral symptoms who are receiving antipsychotic medications, thus taking a novel approach of addressing the neuropsychiatric symptoms that are prevalent and burdensome yet often overlooked (Zhao et al., 2016). Additionally, results are awaited from a phase 1/2 study using human placenta-derived MSCs in probable AD patients over age 50 (NCT02899091).

In summary, current phase 1 and phase 2 clinical trials investigating MSCs and their exosomes for the treatment of AD have shown an excellent safety profile overall, but more extensive phase 2 and 3 studies are required to further characterize their ability to modify the course of AD. A brief summary of clinical trials involving MSCs for AD is presented in Table 1.

**TABLE 1:** Clinical trials investigating MSC-based therapies for AD.

Trial ID, status, trial phase	Study design	Number and type of participants	Cell-type and route of administration	Intervention	Results	References
NCT01297218, Completed, Phase 1	Open label, single arm	9 patients w/probable AD	Allogenic huC-MSCs, Stereotactic Injection	3.0 * 10^6^ or 6.0 * 10^6^ cells	No serious adverse events during 24 month follow-up period. No significant improvement in symptoms	(Kim H. J. et al., 2015)
NCT02054208, Completed, Phase 1/2	Randomized, double blind, placebo-controlled, parallel assignment	46 patients w/probable AD	NEUROSTEM ^®^ (hUC-MSCs), Intraventricular	3 injections of either 1.0 * 10^7^ or 3.0 * 10^7^ cells at 4 week intervals	2 serious adverse events: fever in a low dose PPT, and nausea and vomiting in another low dose PPT. Transient decrease in Aβ and tau was observed, but rose to baseline after 4 weeks.	(Kim H. J. et al., 2021)
NCT03172117, Completed, Phase 1/2	Longitudinal follow-up study	5 patients from trial NCT02054208	N/A	N/A	No further serious adverse events	(Kim H. J. et al., 2021)
NCT04040348, Completed, Phase 1	Open label, single arm	6 patients w/mild-to-moderate probable AD	Allogenic huC-MSCs, Intravenous	4 infusions of 1.0 * 10^8^ cells once every 13 weeks	Not yet published	N/A
NCT03117738, Completed, Phase 1/2	Randomized, double blind, placebo-controlled, parallel assignment	21 patients w/mild-moderate probable AD	AstroStem ^®^ (Autologous AD-MSCs), Intravenous	9 infusions of 2.0 * 10^8^ cells, once every 2 weeks	Serious adverse events in the treatment group include: Diarrhea, acute pulmonary edema, Esophageal Squamous cell carcinoma stage IV. No significant difference observed in cognitive scores.	N/A
NCT02600130, Completed, Phase 1	Randomized, double blind, placebo-controlled, parallel assignment	33 patients w/mild probable AD	Longeveron ^®^ MSCs (allogenic BM-MSCs), Intravenous	1 infusion of either 2.0 * 10^7^ or 1.0 * 10^8^ cells	No treatment-related serious adverse events occurred. No significant change in measures of cognition	(Brody et al., 2023)
NCT02833792, Unknown Status, Phase 2	Randomized, single-blind, placebo-controlled crossover study	40 patients w/mild-to-moderate probable AD	Allogenic hMSCs, Intravenous	1 infusion of 1.5 * 10^6^ cells/kg body Weight	Not yet published	N/A
NCT04954534, Unknown Status, Phase N/A	Open label, single arm	9 patients in control group in trial NCT02054208	NEUROSTEM ^®^ (hUC-MSCs), Intraventricular	3 injections of either 1.0 * 10^7^ or 3.0 * 10^7^ cells, once every 4 weeks	Not yet published	N/A
NCT04482413, Unknown Status, Phase 2b	Randomized, double blind, placebo-controlled, parallel assignment	100 patients w/probable AD	AstroStem ^®^ (Autologous AD-MSCs), Intravenous	9 infusions of 2.0 * 10^8^ cells in 20 mL saline with 30% auto-serum	Not yet published	N/A
NCT04388982, Completed, Phase 1/2	Open label, non-randomized, sequential assignment, dose escalation study	9 patients w/mild-moderate probable AD	Allogenic AD-MSC-derived exosomes, Intranasal Drip	24 administrations of 5, 10, or 20 μg of MSC-Exos. Administered twice a week for 12 weeks.	No adverse events. Improvement in ADAS-Cog and MoCA in medium-dose arm.	(Xie et al., 2023)
NCT02899091, Active, Not Recruiting, Phase 1/2	Randomized, double blind, placebo-controlled, parallel assignment	24 patients w/probable AD	CB-AC-02 (human placental MSCS), Intravenous	Group 1: 1 infusion of 2.0 * 10^8^ cells. Group 2: 2 infusions of 2.0 * 10^8^ cells 4 weeks apart.	–	N/A
NCT06781333, Recruiting, Phase 2	Open label, single arm	8 patients w/probable AD and behavioral symptoms receiving antipsychotic medications	human MSCs, Intravenous	1 infusion of 2.5 * 10^7^ cells	–	N/A
NCT06775964, Recruiting, Phase 1/2	Open label, single arm	12 patients w/late-presymptomatic or prodromal AD	Autologous AD-MSCs, Intravenous	4 infusions of 2.0 * 10^8^ cells at 3 week intervals	–	N/A
NCT06607900, Not Yet Recruiting, Phase 1	multi-center, open label, single arm, basket design	100 patients with AD, PD, MSA, LBD, or FTD	HUC-MSC-sEV-001 (human UC-MSC-derived exosomes), Intranasal	Dose Unspecified	–	N/A

Data in this table was gathered using clinicaltrials.gov (Clinicaltrials.gov, 2025) or through consulting the corresponding reference listed in the table. References are provided only if the clinical trial results have been published. ADAS-Cog, Alzheimer’s Disease Assessment Scale Cognitive Subscale; MoCA, Montreal Cognitive Assessment. Clinicaltrials.gov is not always up to date. Presented data reflect data in corresponding publications where available. Efforts were made to include completed trials as well as those in the “active, not recruiting,” “recruiting,” and “not yet recruiting” phases. Clinical trials with unknown status are included when appropriate.

### 3.2 Parkinson’s disease

Parkinson’s Disease is the second most common neurodegenerative disease behind AD, affecting around 1 in 100 people over 60 years of age and 1 in 25 people over the age of 80 (Pardo-Moreno et al., 2023). PD is classically characterized by progressive motor weakness, tremors, bradykinesia, and muscle rigidity and loss of dopaminergic neurons in the substantia nigra pars compacta (SNpc) (Lev et al., 2003; Kalia and Lang, 2015). Pathologically, this loss of dopaminergic neurons is associated with the deposition of Lewy bodies in the SNpc, which are aggregates of misfolded a-synuclein proteins (Pardo-Moreno et al., 2023). Though it should be noted that the pathology of PD is heterogenous and may feature protein aggregates other than Lewy Bodies. The natural course of PD may begin years before the first characteristic motor symptoms appear, which makes the early diagnosis and treatment of Parkinson’s difficult (Kalia and Lang, 2015).

Inflammation plays a major role in the pathogenesis of PD. In areas of pathology accumulation, microglia and astrocytes are activated and T-cells are recruited to the SNpc, contributing to neuroinflammation (Phani et al., 2012). Activated microglia secrete pro-inflammatory cytokines, ROS, and NO as well as activate the NF-kB signaling pathway, all of which contribute to the downstream death of neurons in the SNpc. The neurodegenerative burden of a-synuclein deposition is increased substantially by the reduction in microglia autophagy in PD. Extracellular a-synuclein inhibits microglia autophagy by interacting with Toll-like receptor-4 (TLR-4) and the p38, AKT-mTOR signaling cascade (Tu et al., 2021), and this loss of autophagic activity is correlated with worsened PD-like symptoms in mouse models (Cheng J. et al., 2020).

Other mechanisms such as dopamine metabolism, microglial autophagy, mitochondrial dysfunction, and even aging itself can contribute to oxidative stress in PD, perpetuating a loop of ROS and reactive nitrogen species generation (Dias et al., 2013). Another self-perpetuating mechanism of dopaminergic neuron death is enacted by monoamine oxidase-B. This enzyme is responsible for decomposing dopamine, released from damaged dopaminergic neurons, into toxic hydrogen peroxide (Kimelberg and Katz, 1986; Inazu et al., 1999; Youdim et al., 2006; Dias et al., 2013).

#### 3.2.1 MSC preclinical trials in PD

Animal models of PD have historically been created through use of toxic molecules like 6-hydroxydopamine (6-OHDA), MPTP, MPP +, paraquat, rotenone, and permethrin (Prasad and Hung, 2020). In 2008, Bouchez et al. used a rat model of PD to show that MSCs could improve motor function when injected into the rat brains (Bouchez et al., 2008). Similarly to preclinical studies in rodent models of AD, MSCs in PD promote microglial autophagy which clears a-synuclein plaques, possibly through the activity of Beclin-1, and enhance cellular viability (Park et al., 2014; Chen H. X. et al., 2020). Furthermore, Schwerk et al. reported that AD-MSCs increased sub-ventricular zone neurogenesis in a rat model of PD, an area of the brain associated with the sleep disturbances observed in early PD (Schwerk et al., 2015).

Similarly to animal models of AD, dextran-coated IONPs have been employed to help target MSC therapies to the substantia nigra. Chung et al. found that loading MSCs with dextran-coated IONPs improved homing to dopaminergic lesions and induced dopaminergic-like differentiation of MSCs in a mouse model of PD (Chung et al., 2018). Use of magnetic resonance-guided focused ultrasound techniques to transiently disrupt the BBB has also shown promising results in improving MSC delivery to the brain in rat models of PD (Wu S. K. et al., 2025).

#### 3.2.2 MSC clinical trials in PD

The first use of MSCs in clinical trials of PD was reported in 2010 by Venkataramana et al. (2010). 7 patients underwent unilateral implantation of autologous BM-MSCs in the sublateral ventricular zone. 3 patients demonstrated continuous clinical improvement with no serious adverse events (Venkataramana et al., 2010). The same research group conducted a similar study with allogenic BM-MSCs transplanted bilaterally in the subventricular zone and reported positive results. Notably, patients in this trial who had PD for less than 5 years experienced greater clinical effects, with more advanced cases showing no clinical improvement (Venkataramana et al., 2012).

More recent clinical trials have expanded on the efficacy and mechanism of action of MSCs in treating PD. A phase 1 dose-escalation study (NCT02611167) using allogenic BM-MSCs reported a lack of serious treatment-related adverse events in addition to a decrease in TNF-α, Chemokine Ligand 22, and an increase in BDNF in the highest dose. This modulation of inflammatory factors was correlated with greater clinical benefit as measured by the UPDRS (Schiess et al., 2021). Preliminary results from a phase 2 trial (NCT04506073) suggest that motor improvements in patients treated with allogenic BM-MSCs are associated with lower levels of serum neurofilament light chain (NfL) (Lemus et al., 2025), a marker of neuronal damage (Gaetani et al., 2019).

Upcoming clinical studies feature alternative administration strategies and MSC treatment formulations. A phase 1/2 trial (NCT03684122) in 10 patients with PD is investigating a combination of hUC-MSCs and hUC-MSC-derived neural progenitor cells administered either intravenously or intrathecally + intravenously. Results on this trial are still forthcoming (Jamali et al., 2021). A currently active phase 2 trial (NCT04995081) is using allogenic AD-MSCs in 60 patients with mild-to-moderate PD. 6 intravenous infusions will be performed at 4-week intervals with the logic that repeated exposure to MSCs will enhance their therapeutic effect. This trial is expected to be completed in 2026. A phase 1 study scheduled to start in 2026 (NCT05094011) will investigate the safety and efficacy of stereotactic intrastriatal AD-MSCs in 9 patients with idiopathic PD. An upcoming phase 1/2 trial (NCT06858254) will investigate a combined intranasal and intravenous approach in patients with either PD or Parkinson’s Disease plus (PPS). Additionally, a phase 1 study scheduled for completion in 2027 (NCT05152394) will report on the efficacy of hUC-MSC-derived exosomes in improving symptoms of PD. A summary of clinical trials for PD using MSCs are presented in Table 2.

**TABLE 2:** Clinical Trials investigating MSC-based therapies for PD.

Trial ID, status, trial phase	Study design	# PPTS	Cell-type and route of administration	Intervention	Results	Citation
NA, Completed, Pilot	Open label, single arm	7 patients w/PD	Autologous BM-MSCs, Unilateral stereotactic injection into subventricular zone	1 injection of 1.0 * 10^6^ cells/kg	3 patients experienced improvement on UPDRS.	(Venkataramana et al., 2010)
NA, Completed, Phase 1	Open label, single arm	8 patients w/PD, 4 patients w/PD plus	Allogenic BM-MSCs, Bilateral stereotactic injection into subventricular zone	1 injection of 2.0 * 10^6^ cells/kg	PD Patients experienced an average of 22% improvement on the UPDRS. No benefit for PD plus patients.	(Venkataramana et al., 2012)
NCT02611167, Completed, Phase 1	Open label, non-randomized, sequential assignment, dose escalation study	20 patients w/idiopathic PD	Allogenic BM-MSCs, Intravenous	1 infusion of 1.0, 2.0, 6.0, or 10.0 * 10^6^ MSCs/kg	No serious adverse events related to the infusion were reported. In Highest dose, TNF-a, and Chemokine ligand 22 decreased and BDNF increased	(Schiess et al., 2021)
NCT04928287, Completed, Phase 2	Randomized, double-blind, placebo-controlled, parallel assignment	24 patients w/early or moderate PD	Autologous AD-MSCs, Intravenous	6 infusions. dosage unspecified	No significant change in MDS-UPDRS. One serious adverse event of Dyspnea in Treatment group	N/A
NCT04506073, Completed, Phase 2a	Randomized, double-blind, placebo-controlled, parallel assignment	45 Patients w/idiopathic PD	Allogenic BM-MSCs, Intravenous	2-3 infusions of 10 * 10^6^ MSCs/kg once every 4 months.	NfL may be a predictor of motor symptom improvement in PD patients treated with MSCs	(Lemus et al., 2025)
NCT03550183, Unknown, Phase 1	Randomized, open label, controlled	20 patients w/PD	Allogenic hUC-MSCs, Intravenous	3 influsions of 10-20 * 10^6^ cells, once every 3 weeks.	Not yet published	N/A
NCT03684122, Unknown, Phase 1/2	Randomized, open label, single arm	10 patients w/PD	Allogenic WJ-MSCs, IV or IV + IC Injection	Group 1: 3 administrations of 40–60 * 10^6^ MSCs via IV + 80–120 * 10^6^ MSCs intrathecally. Group 2: 3 administrations of 40–60 * 10^6^ MSCs via IV + 12 * 10^6^ NSCs intrathecally.	Not yet published	(Jamali et al., 2021)
NCT04995081, Active, Not Recruiting, Phase 2	Randomized, double-blind, parallel assignment	60 patients w/mild-to-moderate PD	Allogenic AD-MSCs, Intravenous	6 infusions at 4 week intervals, dosage unspecified	–	N/A
NCT05152394, Recruiting, Phase 1	Open label, single arm	20 Patients w/PD	Allogenic hUC-MSC-derived exosomes, Intranasal	2 administrations of ∼800 billion MSC-exos	–	N/A
NCT05094011, Not Yet Recruiting, Phase 1	Open label, sequential assignment, dose escalation study	9 patients w/idiopathic PD	MitoCell ^®^ (Autologous AD-MSCs), Stereotactic Intrastriatal Implantation	1 injection of 3.0 *10^7^ or 1.0 * 10^8^ cells/hemisphere	–	N/A
NCT06858254, Not Yet Recruiting, Phase 1/2	Randomized, single blind, crossover assignment	60 patients w/PD or PPS	Autologous BM-MSCs, Intravenous and Intranasal	Dose Unspecified	–	N/A

Data in this table was gathered using clinicaltrials.gov (Clinicaltrials.gov, 2025) or through consulting the corresponding reference listed in the table. References are provided only if the clinical trial results have been published. Note: clinicaltrials.gov is not always up to date. Presented data reflect data in corresponding publications where available. PPS = Parkinson’s Plus. NfL = Neurofilament light chain. UPDRS = Unified Parkinson’s Disease Rating Scale. MDS-UPDRS = Movement Disorder Society—Unified Parkinson’s Disease Rating Scale. Efforts were made to include completed trials as well as those in the “active, not recruiting,” “recruiting,” and “not yet recruiting” phases. Clinical trials with unknown status are included when appropriate.

### 3.3 Amyotrophic lateral sclerosis

Amyotrophic Lateral Sclerosis is a neurodegenerative disease that typically presents late in life and is characterized by upper and lower motor neuron dysfunction, which culminates in a number of symptoms, including muscle weakness, dysphagia, dysarthria, respiratory muscle weakness, and autonomic dysfunction, though there is significant phenotypic variation (Feldman et al., 2022). The most common causative genes are *C9orf72, SOD1, TARDBP*, and *FUS* (Chia et al., 2018), but the pathogenesis is considered to be a complex interplay of genetic and environmental factors (Feldman et al., 2022).

Given the genetic and environmental heterogeneity of ALS, the pathophysiologic mechanisms are similarly heterogenous. Pathologically, ALS is characterized by impairment of RNA metabolism, proteostasis, autophagy, cytoskeletal structure and trafficking, and mitochondrial function (Morgan and Orrell, 2016; Nguyen et al., 2018; Feldman et al., 2022), as well as neuroinflammation (Beers and Appel, 2019). One of the dominant pathologic actors in ALS is the TAR DNA-binding protein 43 (TDP-43) produced by the *TARDBP* gene. TDP-43 regulates many processes related to RNA processing, and defects in this protein cause cellular damage through cytotoxic aggregation in the cytoplasm and disrupted RNA metabolism. Fused in sarcoma (FUS) proteins, produced by the *FUS* gene are another common effector protein involved with nucleic acid processing that co-localizes with TDP-43 inclusions. Together, intracellular TDP-43 and FUS aggregates disrupt normal RNA and protein processing, leading to propagation of mis-folded proteins and cellular toxicity (Morgan and Orrell, 2016). Furthermore, TDP-43 and FUS can perpetuate motor neuron degeneration by inducing prion-like spread of mis-folded superoxide dismutase 1 (SOD1) protein, contributing to the progressive spread of ALS (Pokrishevsky et al., 2016). The neuronal dysfunction caused by mis-folded protein aggregates can precipitate neuroinflammatory mechanisms such as glutamate excitotoxicity, mitochondrial oxidative stress, and glial activation (Liu and Wang, 2017), all of which further contribute to neurodegeneration.

#### 3.3.1 MSC preclinical trials in ALS

Early preclinical trials studying MSCs in models of ALS primarily utilized SOD1-G93A mice (Zhao et al., 2007). These mice carry a pathogenic form of the SOD1 gene which leads to the development of ALS (Chia et al., 2018). MSCs have been shown to slow neuronal and axonal degeneration in ALS, slow motor function deterioration, extend lifespan, and attenuate microglial and astrocyte activation in SOD1-G93A mice (Zhao et al., 2007; Vercelli et al., 2008; Kim H. et al., 2010). Though these studies were the first to demonstrate the efficacy of MSCs in treating ALS, in both experiments the MSC treatment was administered in pre-symptomatic mice. Early intervention before symptoms appear is uncommon in the traditional healthcare setting because symptoms onset is slow and non-specific (Feldman et al., 2022).

Studies performed in symptomatic animal models of ALS yielded similar results (Boucherie et al., 2009; Uccelli et al., 2012; Marconi et al., 2013), suggesting that MSCs are effective in treating ALS even when administered after symptom onset. Exosomes derived from MSCs have also shown promising neuroprotective benefit both *in vitro* and *in vivo* models of ALS (Bonafede et al., 2016; Bonafede et al., 2020).

#### 3.3.2 MSC clinical trials in ALS

The first study to demonstrate that MSC treatment for ALS was feasible in human subjects was reported on by Mazzini et al. in 2003. The authors reported no adverse events in 7 patients transplanted intrathecally with autologous BM-MSCs (Mazzini et al., 2003). However, due to the small sample size, no conclusions could be made as to the efficacy of the treatment. Two follow-up clinical trials were conducted by Mazzini to further investigate the safety and characterize the effects of MSC treatment in ALS patients (Mazzini et al., 2010; Mazzini et al., 2012). The first study in 10 patients with ALS treated intrathecally with autologous BM-MSCs reported no serious treatment-related adverse events (Mazzini et al., 2010). The second study was a longitudinal follow-up study in the 19 patients enrolled in the first two phase 1 clinical trials. After a 9-year follow-up period the treated patients demonstrated no abnormal structural changes in the brain or spinal cord and no decline in psychological status. However, no clinical improvement was noted (Mazzini et al., 2012). Despite lack of clear therapeutic benefit, these studies were the first to prove that MSC treatment for ALS is safe and feasible both in the short- and long-term.

Another early phase clinical trial (NCT00855400) conducted by Blanquer et al. adopted the approach used by Mazzini et al. to investigate autologous BMMNCs in the treatment of ALS (Blanquer et al., 2012). 11 ALS patients were injected intrathecally BMMNCs following laminectomy. In 1 year of follow up no serious treatment-related adverse events were recorded and no increase in clinical rate of decline was reported. Histological examination of the spinal cord of 4 patients who passed away due to causes unrelated to treatment revealed a higher density of motoneurons in regions of the spinal cord injected with BMMNCs, and these motoneurons exhibited fewer intracellular TDP-43 deposits. Interestingly, a number of surviving motoneurons were surround by CD90 + cells, and these motoneurons displayed no TDP-43 reactivity, suggesting that CD90 + cells may exert a neuroprotective effect in ALS by preventing TDP-43 aggregation or enhancing clearance (Blanquer et al., 2012). Overall, this study provided evidence for the neuroprotective effect of BMMNCs in ALS in humans.

Not long after, a phase 1/2 and 2a clinical trial (NCT01051882 and NCT01777646) conducted by Petrou et al. in 28 patients administered NurOwn^®^ cells (autologous BM-MSC-NTF cells) both intramuscularly and intrathecally reported that the treatment was safe and modestly effective in slowing motor decline (Petrou et al., 2016). The cells used in this trial had been specifically cultured to produce neurotrophic factors to promote cell survival and endogenous mechanisms of recovery (Gothelf et al., 2014). The results of a larger phase 2 trial enrolling 48 patients indicate that treatment with NurOwn^®^ cells was capable of delaying the clinical course of ALS in a predefined rapidly progressing subset of patients (Berry et al., 2019).

Petrou would conduct another (NCT04821479) focused on repeat injection of autologous BM-MSCs. 20 patients with ALS were administered up to 4 intrathecal injections of autologous BM-MSCs. No serious adverse events were reported, and the treatment was found to be effective after just 1 injection (Petrou et al., 2021). There was significant participant drop-off and loss to follow up, but patients who remained in the study experienced a significant improvement in the rate of progression of ALS when measured using the ASLFRS-R (Petrou et al., 2021).

A phase 3 study (NCT03280056) investigating NurOwn^®^ cells was recently completed. 196 patients with ALS were administered either the MSC treatment or placebo via intrathecal injection. The treatment was well-tolerated, but the primary efficacy endpoint, change in ALSFRS-R progression compared to controls at 28 weeks, was not statistically significant. However, MSC treatment reduced markers of neuroinflammation and neural damage while increasing levels of neurotrophic factors in the CSF (Cudkowicz et al., 2022). It was theorized that the therapy may be more effective in treating early/mild ALS, so a follow-up phase 3 clinical trial (NCT06973629) in early symptomatic ALS and moderate ALS was initiated and is scheduled to be completed in 2028. Another phase 3 trial (NCT04745299) investigating autologous BM-MSCs is currently in progress and scheduled to be completed in 2026. This study plans to enroll 115 patients w/either familial or sporadic ALS. Patients in the single cycle administration group will be given two injections of MSCs within 26 days, and patients in the multiple administrations group will be given 2 injections in 26 days followed by 3 repeat injections every 3 months. Additionally, each patient will be administered riluzole for the study duration. These larger trials will provide data on the efficacy of MSC treatments for ALS.

Two upcoming registered clinical trials plan to study the MSC secretome in ALS patients. The phase 1 basket-design clinical trial (NCT06607900) discussed earlier will include an ALS arm. Another phase 1/2 placebo-controlled clinical trial (NCT06598202) will use hUC-MSC exosomes to treat early-stage ALS (< 2 years) in 38 patients. This study is expected to be completed in 2026. Table 3 includes a summary of completed and upcoming clinical trials of MSCs for PD.

**TABLE 3:** Clinical trials investigating MSC-based therapies for ALS.

Trial ID, status, phase	Study design	# PPTS	Cell-type and route of administration	Intervention	Results	Citation
NA, Completed, Phase 1	Open label, single arm	7 patients w/sporadic ALS	Autologous BM-MSCs, Intrathecal	1 injection of between 7 and 152 million cells	No serious adverse events. No evidence of change in spinal cord volume or abnormal cell proliferation.	(Mazzini et al., 2003)
NA, Completed, Phase 1	Open label, single arm, dose escalation study	10 patients w/sporadic ALS	Autologous BM-MSCs, Intrathecal	1 injection of ∼75 * 10^6^ cells (range: 11–120 *10^6^)	No immediate or delayed treatment-related adverse events or toxicity. No structural changes or tumors in brain or spinal cord on MRI.	(Mazzini et al., 2010)
NA, Completed, Phase N/A	Longitudinal follow-up	19 patients w/ALS treated with autologous BM-MSCs	NA	NA	No abnormal structural changes or psychological deterioration. No clinical benefit observed	(Mazzini et al., 2012)
NCT00781872, Completed, Phase 1/2	Open label, single arm	19 patients w/ALS. 15 patients w/MS	Autologous BM-MSCs, Intravenous and Intrathecal	Group 1: 54.7 * 10^6^ cells/kg intrathecally (n = 19). Group 2: 54.7 *10^6^ cells/kg intrathecally + 23.4 * 10^6^ cells intravenously (N = 9) (Only ALS group)	MSC treatment is safe and feasible in ALS and MS and induced immunomodulatory effects	(Karussis et al., 2010)
NCT00855400, Completed, Phase 1/2	Open label, single arm	11 patients w/ALS	Autologous BMMNCs, Intraspinal	Laminectomy followed by intraspinal transplantation of ∼462 * 10^6^ BMMNCs (range: 138–602.87 * 10^6^).	No serious adverse events observed. Pathologic spinal cord specimens demonstrated greater number of motoneurons in regions treated with MSCs.	(Blanquer et al., 2012)
NCT01609283, Completed, Phase 1	Open label, single arm	27 patients w/ALS	Autologous AD-MSCs, Intrathecal	Group 1: 1 injection of 1.0 * 10^7^ cells. Group 2: 1 injection of 5.0 * 10^7^ cells. Group 3: 2 injections of 5.0 * 10^7^ cells at 1 month intervals. Group 4: 1 injection of 1.0 * 10^8^ cells. Group 5: 2 injections of 1.0 * 10^8^ cells at 1 month intervals.	Treatment found to be safe and feasible. No significant clinical delay in ALS progression.	(Staff et al., 2016)
NCT01051882*, Completed, Phase 1/2	Open label, non-randomized, parallel assignment	12 patients with ALS	NurOwn ^®^ cells (Autologous BM-MSC-NTF cells), Intramuscular or Intrathecal	24 * 10^6^ cells intramuscularly, or 60 * 10^6^ cells intrathecally.	Treatment safe and well tolerated overall. IT treated cohorts of both studies experienced slowing of rate of progression in ALSFRS-R and forced vital capacity.	(Petrou et al., 2016)
NCT01777646*, Completed, Phase 2a	Open label, dose escalation study	14 patients w/ALS	NurOwn ^®^ cells (Autologous BM-MSC-NTF cells), Intramuscular + Intrathecal	Group 1: 1.0 * 10^6^ cells/kg IT + 24 * 10^6^ cells IM. Group 2: 1.5 * 10^6^ cells/kg IT + 36 * 10^6^ cells IM. Group 3: 2.0 * 10^6^ cells/kg IT + 48 * 10^6^ cells IM.	Treatment safe and well tolerated overall. IT treated cohorts of both studies experienced slowing of rate of progression in ALSFRS-R and forced vital capacity.	(Petrou et al., 2016)
NCT02017912, Completed, Phase 2	Randomized, double blind, placebo controlled, parallel assignment	48 patients w/ALS	NurOwn ^®^ cells (Autologous BM-MSC-NTF cells), Intramuscular + Intrathecal	Single combined administration of 24 IM injections (48 * 10^6^ cells) + 125 * 10^6^ cells Intrathecally	No treatment-related serious adverse events occurred. Slight delay in clinical course for treated participants.	(Berry et al., 2019)
NCT04821479, Completed, Phase 1/2	Open label, single arm	20 patients w/ALS, ALSFRS-R > 15 at screening	Autologous BM-MSCs, Intrathecal	4 injections of 1.0 * 10^6^ cells/kg, once every 3 months	No serious adverse events were observed. Significant delays in ALS progression in 15/19 patients via ALSFRS-R.	(Petrou et al., 2021)
NCT03280056, Completed, Phase 3	Randomized, double blind, placebo controlled, parallel assignment	196 patients w/ALS; ALSFRS-R > 25	NurOwn ^®^ (Autologous BM-MSC-NTF cells), Intrathecal	3 injections of 100–125 * 10^6^ cells once every 8 weeks.	Primary endpoint not met. No significant improvement in ALSFRS-R in treatment group compared to control. Significant imporvements in cerebrospinal markers of neuroinflammation observed in MSC group.	(Cudkowicz et al., 2022)
NCT01363401, Completed, Phase 1/2	Randomized, open-label, parallel assignment	72 patients w/ALS < 5 years	Autologous BM-MSCs, Intrathecal	riluzole combined with 2 IT injections of 1.0 * 10^6^ cells/kg	slowed progression of ALSFRS-R in treated group. Reduced proinflammatory/increased anti-inflammatory cytokines in treated group	(Oh et al., 2018)
NCT047452999, Active, Not Recruiting, Phase 3	Randomized, double blind, placebo controlled, parallel assignment	115 patients w/ALS < 2 years	Lenzumestrocel ^®^ (Autologous BM-MSCs), Intrathecal	2 injections, or 2 injections followed by 3 repeat injections. Dosage Unspecified	-	N/A
NCT06598202, Recruiting, Phase 1/2	Randomized, double blind, placebo controlled, sequential assignment, dose escalation	38 patients w/ALS < 2 years	hUC-MSC-sEV-001 ^®^ (hUC-MSC-derived exosomes), Intranasal	Intranasal hUC-MSC exosomes. Low dose group, middle dose group, high dose group. Twice a week for 2 weeks. Dosage unspecified	-	N/A
NCT06973629, Not Yet Recruiting, Phase 3	Randomized, double blind, placebo-controlled, parallel assignment	200 patients w/early symptomatic ALS < 2 years	Debamestrocel-NurOwn ^®^ (Autologous-MSC-NTF cells), Intrathecal	3 injections of MSCs, once every 8 weeks. Dosage unspecified	-	N/A
NCT06910384, Not Yet Recruiting, Phase 2	Open label, single arm	9 patients w/ALS	Autologous BM-MSCs, Intravenous	4 infusions, once every 12 weeks. Dosage unspecified	-	N/A

Data in this table was gathered using clinicaltrials.gov (Clinicaltrials.gov, 2025) or through consulting the corresponding reference listed in the table. References are provided only if the clinical trial results have been published. Note: clinicaltrials.gov is not always up to date. Presented data reflect data in corresponding publications where available.

*The results of NCT01051882 and NCT01777646 were published in the same journal article, Petrou et al., 2016. IM, Intramuscular; IT, Intrathecal; ALSFRS, ALS functional rating scale; ALSFRS-R, ALS functional rating scale revised. Efforts were made to include completed trials as well as those in the “active, not recruiting,” “recruiting,” and “not yet recruiting” phases. Clinical trials with unknown status are included when appropriate.

### 3.4 Multiple sclerosis

Multiple Sclerosis is a demyelinating disease that affects individuals typically in their early adulthood and is more prevalent in women. The most common form of MS is relapse-remitting MS, which is characterized by periods of neurologic symptoms, caused by acute CNS inflammation, followed by periods of quiescence. The exact symptoms depend on the location of the inflammation occurs with episodes typically self-resolving (Thompson et al., 2018). Patients may also present with a steadily progressive form of MS known as primary progressive MS (PPMS). There exists a tertiary category known as secondary progressive MS (SPMS), which presents as RRMS followed by a steadily progressive phenotype similar to PPMS (Miller and Leary, 2007). The major symptoms of MS include optic neuritis due to inflammation of the optic nerve, sensory symptoms, fatigue, pain, decreased mobility, muscle spasticity, and bowel and bladder symptoms (Kister et al., 2013). MS is also associated with cognitive deficits and neuropsychiatric symptoms like depression, anxiety disorders, and even personality changes (Kister et al., 2013; Silveira et al., 2019).

MS affects over 2.3 million people globally. It has been associated with several potentially causative factors. Environmental risk factors include vitamin D deficiency, early life obesity, and smoking (Thompson et al., 2018). Genetic risk factors include pathogenic variants in the HLA locus, which produces the major histocompatibility complex (MHC) protein. The HLA-DRB1*15:01 allele confers particularly high risk (Patsopoulos et al., 2013). Pathologically, MS is characterized by axonal and neuronal loss, demyelination, and gliosis. Many of the same mechanisms of CNS injury are at play in MS as in other conditions, such as astrocyte and microglia activation, glutamate excitotoxicity, and ionic imbalances, but the primary mechanism of pathogenesis in MS is autoimmunity (Thompson et al., 2018). CD4 and CD8 and Th17 T-cells react to components of the myelin sheaths and cause demyelination. B-cells are also implicated through the secretion of cytokines and antigen presentation to T-cells (Thompson et al., 2018). Current treatments for MS focus on limiting T and B-cell proliferation, impairing the auto-reactivity of T and B-cells, and inducing their differentiation to anti-inflammatory phenotypes (Thompson et al., 2018; Dobson and Giovannoni, 2019; Gugliandolo et al., 2020).

#### 3.4.1 MSC preclinical trials in MS

Preclinical studies of MS have historically used an experimental autoimmune encephalitis (EAE) model. EAE is an artificial autoinflammatory condition created by sensitizing the host immune system to autoantigens, thereby activating the immune system against itself (Constantinescu et al., 2011). The EAE model has been used for decades to study a variety of autoinflammatory nervous system disorders (Rivers et al., 1933; Rivers and Schwentker, 1935).

The first preclinical trial studying the potential of MSCs in treating EAE was conducted in Zappia et al. (2005). This study induced EAE using myelin oligodendrocyte glycoprotein 35-55 (MOG35-55). EAE mice were treated intravenously with BM-MSCs. Treated mice experienced profound reduction in EAE severity without inducing tumorigenicity or immunodeficiency. Evidence collected on T-cell activity during the study demonstrated significant reduction in T-cell proliferation when treated with BM-MSCs (Zappia et al., 2005), which is consistent with evidence reported from previous *in vitro* models (Bartholomew et al., 2002). It should be noted that the mice in this study were administered BM-MSCs at either the onset of symptoms or during the peak of symptom severity, as opposed to after resolution of symptoms, a method which has become standard practice (Zappia et al., 2005; Kurte et al., 2015).

Further analysis of the immunomodulatory effects of MSCs in subsequent preclinical models of MS revealed that MSCs reduce T-cell secretion of TNF-α and IFN-γ and reduce T-cell proliferation upon contact with antigen (Gerdoni et al., 2007). Furthermore, BM-MSC transplantation in an EAE model of MS reduced mortality to 0% (Kassis et al., 2008). These results were consistent with those reported by Zappia et al. (2005). These studies together provided strong evidence that MSCs could serve as a potential treatment for MS in humans.

#### 3.4.2 MSC clinical trials in MS

MSCs have been shown to be moderately effective in treating MS in human clinical trials. A meta-analysis of the clinical trials involving MSCs in treating MS conducted in 2023 showed that about 40% of MS patients treated with MSCs saw improvement. 32.8% remained stable, and 18.1% worsened. Headaches and fevers were the most common side effects. These data suggest that MSC therapy for MS is overall safe in humans and shows therapeutic promise (Islam et al., 2023).

The first clinical trials in humans investigating the potential of MSCs for treating MS were conducted in the mid-2000s. In the first study investigators treated 5 patients with SPMS with autologous BM-MSCs via intrathecal route. The treatment was deemed safe, but few patients experienced clinical benefit as measured by the Expanded Disability Scale Score (EDSS) (Mohyeddin Bonab et al., 2005). A follow up study was conducted in 10 patients with PPMS or SPMS. These patients were also treated with autologous BM-MSCs, but only a minority experienced clinical benefit (Mohyeddin Bonab et al., 2007). These studies provided the first in human evidence for the feasibility of MSC therapy for patients with MS.

A phase 2 clinical trial based on these initial studies was later conducted by the same research group (Bonab et al., 2012). In this study, 25 patients with MS were injected intrathecally with autologous BM-MSCs and monitored for 1 year. No serious adverse events were reported, but only 4 patients experienced clinical improvement in the EDSS, whereas 6 patients experienced in a decline in the EDSS. Additionally, 6 patients demonstrated new or worsening lesions on magnetic resonance imaging (MRI) (Bonab et al., 2012).

The results of another early phase 1 study published in 2010 confirmed the safety of autologous BM-MSCs for treating MS (Yamout et al., 2010). 10 patients with advanced MS were administered autologous BM-MSCs intrathecally and followed up for 1 year. EDSS metrics revealed improvement in 5 of the 7 patients, but MRI indicated worsening lesions in 5 of the 7 patients as well (Yamout et al., 2010), consistent with other trials (Bonab et al., 2012). The only serious adverse event was transient encephalopathy with seizures in 1 patient (Yamout et al., 2010).

Karussis et al. published the results of a phase 1/2 clinical trial (NCT00781872) in the same year which featured both an ALS treatment group (*n* = 19) and an MS treatment group (*n* = 15) (Karussis et al., 2010). They transplanted 15 MS patients with BM-MSCs both intrathecally and intravenously and observed no serious adverse events. The mean EDSS score of patients improved and markers of immune cell activation and proliferation were reduced. Notably, peripheral CD4 + and CD25 + regulatory T-cell populations in the blood were increased following MSC administration (Karussis et al., 2010). This is consistent with evidence from preclinical trials that MSCs target and regulate the adaptive immune system (Zappia et al., 2005; Gerdoni et al., 2007).

A phase 1/2 clinical trial (NCT00395200) published in 2012 treated 10 patients with SPMS and evidence of optic nerve involvement with autologous BM-MSCs. No serious adverse events were reported from intravenous administration. The Investigators found that on average patients treated with autologous BM-MSCs experienced higher visual acuity scores and visual evoked response latency compared to baseline in addition to increased optic nerve area (Connick et al., 2012). These results suggest that MSCs can improve visual symptoms in MS.

Two clinical trials conducted by Harris et al. would investigate the use of MSC-derived neural progenitor cells (MSC-NP) in patients with progressive MS. MSC-NP cells are a subtype of MSC which exhibit neural characteristics (Harris et al., 2012). In 2016, the results of a pilot study in 6 patients with MS were reported, which showed promising safety and efficacy results. No serious adverse events were reported, and 4 of the 6 patients experienced improvement on the EDSS relative to baseline (Harris et al., 2016). In 2018 the results of a full phase 1 study (NCT01933802) in 20 patients with progressive MS were reported. The researchers administered 3 intrathecal doses of MSCs and observed no serious treatment-related adverse events and reported measurable improvements in several patients in EDSS scores, muscle group strength, and bladder symptoms (Harris et al., 2018).

The results of a large phase 2 trial (NCT02239393) conducted by Uccelli et al. were reported in 2021. 144 patients with MS were treated intravenously with autologous BM-MSCs. No treatment-related serious adverse events were reported, but ultimately the trial did not meet the primary efficacy endpoint of reducing gadolinium-enhancing lesions (GEL) on MRI (Uccelli et al., 2021). Another phase 2 trial (NCT03799718) investigated the safety of NurOwn^®^ cells in treating PPMS and SPMS. 18 patients with PPMS or SPMS were treated with 3 intrathecal injections of NurOwn^®^ cells (17 patients received all 3 injections). 7 serious treatment-related adverse events were reported. Several patients observed improvements in motor symptoms (Cohen et al., 2023), but larger studies with control comparison are needed to further investigate these cells.

Though most of the clinical trials utilizing MSCs to treat MS have employed BM-MSCs, other sources of MSCs have been investigated. Multiple studies (NCT05116540, NCT0105647) have employed AD-MSCs in clinical trials, but despite their good safety profiles, these trials have failed to demonstrate significant clinical improvement (Fernández et al., 2018). Additionally, several trials have investigated hUC-MSCs or placenta-derived MSCs for MS with mixed results (Li et al., 2014; Lublin et al., 2014).

There are several upcoming clinical trials for MSCs in MS. A phase 1/2 clinical trial (NCT04749667) is currently active and aims to enroll 18 patients with primary or SPMS. These patients will be administered autologous BM-MSCs via intrathecal injection. Two clinical trials are in the recruiting phase. The first is a phase 1 study (NCT05003388) which will investigate the safety of allogenic hUC-MSCs in patients with MS. The second is a phase 1/2 trial (NCT05532943) which will recruit 41 patients with RRMS or SPMS. Patients will be administered allogenic hUC-MSCs through intravenous infusion day 0 then again 28 days later via intrathecal infusion. Both studies are expected to be completed in 2025 and 2026 respectively.

The evidence that has been reported from these clinical trials clearly shows the safety of multiple types of MSCs for treating both relapse-remitting and progressive MS, but the current lack of phase 3 clinical trials indicates the need for larger, randomized, placebo-controlled trials to further characterize the efficacy and mechanism of action of MSCs in MS. Table 4 summarizes the findings of clinical trials studying MSCs for therapeutic use in MS.

**TABLE 4:** Clinical trials investigating MSC-based therapies for MS.

Trial ID, status, phase	Study design	# PPTS	Cell-type and route of administration	Intervention	Results	Citation
NA, Completed, Phase 1	Open label, single arm	5 patients w/SPMS, EDSS < 6	Autologous BM-MSCs, Intrathecal	1 injection of 6.0 * 10^6^ cells	Treatment deemed safe and feasible. 3 patients experienced mild improvement on EDSS	(Mohyeddin Bonab et al., 2005)
NA, Completed, Phase1	Open label, single arm	10 patients w/SPMS or PPMS refractory to disease modifying agents. EDSS < 6	Autologous BM-MSCs, Intrathecal	1 injection of 8.73 * 10^6^ cells	1 patients experienced improvement in EDSS by 2.5 points. 7 patients demonstrated no change in lesions on MRI after 1 year.	(Mohyeddin Bonab et al., 2007)
NA, Completed, Phase 1	Open label, single arm	10 patients w/refractory MS. EDSS 4–7.5	Autolgoous BM-MSCs, Intrathecal	1 injection of ∼32–52 * 10^6^ cells.	1 serious adverse event. 5 patients experienced improved EDSS by 0.5–1.0.	(Yamout et al., 2010)
NCT00781872, Completed, Phase 1/2	Open label, single arm	15 patients w/MS, 19 patients w/ALS	Autologous BM-MSCs, Intravenous + Intrathecal	Group 1: 63.2 * 10^6^ cells IT (n = 15). Group 2: 63.2 *10^6^ cells IT + 24.5 * 10^6^ cells IV (N = 5).*	No major adverse events. Mean EDSS score improved during follow-up period. Increase in CD4 +, CD25 + Regulatory T-cells and decrease in lymphocyte proliferation	(Karussis et al., 2010)
NCT00395200, Completed, Phase 1/2	Open label, single arm	10 patients w/MS and history of optic nerve damage. EDSS between 2 and 6.5	Autologous BM-MSCS, Intravenous	1 infusion of 1.1–2.0 * 10^6^ cells/kg	No Serious adverse events were recorded. Clinical improvements in visual acuity and visual evokes response latency and increase in optic nerve area were observed	(Connick et al., 2012)
NA, Completed, Phase 2	Open label, single arm	25 patients w/SPMS or PPMS refractory to disease modifying agents. EDSS between 4 and 6	Autologous BM-MSCs, Intrathecal	1 injection of 29.5 * 10^6^ cells	4 patients experienced improved EDSS while 6 deteriorated. 6 patients showed new GELs on MRI.	(Bonab et al., 2012)
NA, Completed, Phase 1b	Randomized, double blind, placebo controlled, parallel assigment	16 patients w/RRMS or SPMS	Allogenic Placenta-derived MSCs, Intravenous	Low dose: 150 * 10^6^ cells. high dose: 600 * 10^6^ cells.	Treatment safe and well-tolerated. No paradoxical worsening of MS. Inconsistent improvement across patients.	(Lublin et al., 2014)
NA, Completed, Phase 2	Randomized	23 patients w/RRMS or SPMS. EDSS 4–8	Allogenic hUC-MSCs, Intravenous	3 infusions of 4.0 * 10^6^ cells/kg, once every 2 weeks	Slight decrease in average EDSS score and relapse rate compared to controls	(Li et al., 2014)
NA, Completed, Pilot	Open label, single arm, dose escalation	6 patients with PPMS or SPMS. EDSS > 6.5	Autologous BM-MSC-NPCs, Intrathecal	2–5 injections of between 5.0 * 10^3^ cells and 1.6 * 10^7^ cells.	No serious adverse events noted during 7.4 years of follow up. 4 patients experienced improvements on EDSS relative to baseline.	(Harris et al., 2016)
NCT00813969, Completed, Phase 1	Open label, single arm	24 patients w/RRMS or SPMS. EDSS 3.0–6.5	Autolgoous BM-MSCs, Intravenous	1 infusion of 1.0 to 2.0 * 10^6^ cells/kg	Treatment was well-tolerated without treatment-related severe or serious adverse events	(Cohen et al., 2018)
NCT01056471, Completed, Phase 1/2	Randomized, double blind, placebo-controlled, parallel assignment	34 patients w/SPMS. EDSS between 5.5 and 9	Autologous AD-MSCs, Intravenous	Single infusion of 1.0 * 10^6^ cells/kg or 4.0 * 10^6^ cells/kg	Treatment found to be safe and feasible, but no overall clinical benefit was observed.	(Fernández et al., 2018)
NCT02403947, Terminated, Phase 1/2	Randomized, double blind, placebo-controlled, crossover assignment	1 patient w/RRMS	Autologous BM-MSCs, Intravenous	1 infusion of up to 2 * 10^6^ cells/kg	–	(Uccelli et al., 2019)
NCT01933802, Completed, Phase 1	Open label, single arm	20 patients w/PPMS or SPMS. EDSS > 3	Autologous BM-MSC-NP cells, Intrathecal	3 injections of up to 10* 10^6^ cells at 3 months intervals	No treatment-related serious adverse events were observed. A number of patients experienced improvements in EDSS scores, muscle group strength, and bladder symptoms.	(Harris et al., 2018)
NCT02166021, Completed, Phase 2	Randomized, double-blind, placebo controlled, crossover assignment	48 patients w/PPMS or SPMS. EDSS 3.5 and 6.5	Autologous BM-MSCs, Intravenous or Intrathecal	Group 1A: 2 IT doses of 15.0 * 10^6 cells/kg at 6 months intervals. Group 1B: 1 IT dose of 15.0 * 10^6 cells/kg then placebo at 6 months. Group 2A: 2 IV doses of 15.0 * 10^6 cels/kg at 6 month intervals. Group 2B: 1 IV dose of 15.0 * 10^6 cell/kg then placebo at 6 months.	No serious treatment-related adverse events reported. Both MSC treated groups showed high rates of no disease activity compared to controls. MSC treatment also improved other neurological assessment scores.	(Petrou et al., 2020)
NCT04823000, Completed, Phase 1/2	Open label, single arm	24 patients w/active progressive MS. EDSS 5–7.5	Autolgoous BM-MSCs, Intrathecal + Intravenous	Upto 9 administrations of 1.0 * 10^6^ cells/kg IT and 1.0 * 10^6^ cells/kg IV at 6–12 month intervals.	No serious treatment related adverse events occurred. Clinical benefits were observed in those who had more than 2 injections	(Petrou et al., 2021)
NCT02239393, Completed, Phase 2	Randomized, crossover, double-blind, placebo-controlled	144 patients w/RRMS, PPMS, or SPMS. EDSS 2.5–6.5	Autologous BM-MSCs, Intravenous	1 infusion of up to 2.0 * 10^6^ cells/kg	No serious adverse events related to the treatment. No change in GELs on MRI.	(Uccelli et al., 2021)
NCT03799718, Completed, Phase 2	Open label, single arm	23 patients w/PPMS or SPMS. EDSS 3–6.5	NurOwn ^®^ (Autologous BM-MSC-NTF) cell, Intrathecal	3 Intrathecal injections of 100–125 * 10^6^ cells at 8 week intervals.	No treatment related adverse events occurred. In 3 participants motor improvements were observed.	(Cohen et al., 2023)
NCT05116540, Completed, Phase 2	Randomized, double-blind, placebo controlled, parallel assignment	24 patients w/RRMS > EDSS 3.0–6.5	Autologous AD-MSCs, Intravenous	6 infusions of AD-MSCs, once every 4 weeks. Dosage unspecified.	Treatment group improved in both physical health and mental health composite scores. 2 serious adverse events in the treatment group were observed, affecting the same subject.	N/A
NCT04749667, Active, Not Recruiting, Phase 1/2	Randomized, double blind, placebo controlled, crossover assignment	18 patients w/PPMS or SPMS. EDSS 4–7	Autologous BM-MSCs, Intrathecal	1 injection. Dosage unspecified	–	N/A
NCT05532943, Recruiting, Phase 1/2	Randomized, double blind, parallel assignment, placebo-controlled	41 patients w/RRMS or SPMS. EDSS 2–6.5	Allogenic hUC-MSCs, Intravenous + Intrathecal	Intravenous infusion followed by intrathecal injection 28 days later. Dosage unspecified	–	N/A
NCT05003388, Recruiting, Phase 1	Open label, single arm	15 patients w/MS	Allogenic hUC-MSCs, Intravenous	1 infusion of 1.0 * 10^8^ cells	–	N/A

Data in this table were gathered using clinicaltrials.gov (Clinicaltrials.gov, 2025) or through consulting the corresponding reference listed in the table. References are provided only if the clinical trial results have been published. Note: clinicaltrials.gov is not always up to date. Presented data reflect corresponding publications where available. RRMS, Relapse-remitting MS; SPMS, Secondary progressive MS; PPMS, Primary Progressive MS; EDSS, Extended Disability Scale Score; GELs, Gadolinium-Enhancing Lesions; IV, Intravenous; IT, Intrathecal; BM-MSC-NP cells, bone marrow mesenchymal stem cell-derived neural progenitor cells.

*MS groups only. Efforts were made to include completed trials as well as those in the “active, not recruiting,” “recruiting,” and “not yet recruiting” phases. Clinical trials with unknown status are included when appropriate.

### 3.5 Stroke

Stroke describes a rapid-onset neurological deficit caused by anomalies in the cerebral vasculature, including small vessel disease, vascular hemorrhage, and cardioembolism (Hilkens et al., 2024). It is one of the leading causes of death and disability in the world. In 2021 the prevalence of stroke was estimated to 93.8 million and the incidence of stroke 11.9 million. The incidence of stroke has only continued to rise in recent years with prevalence expected to nearly double by 2050 (Feigin et al., 2024). Of the two subtypes of stroke (ischemic and hemorrhagic), ischemic stroke is more common and occurs when blood vessels in the brain are blocked, resulting in acute loss of neurologic function due to neuronal damage (Hilkens et al., 2024).

The secondary injury following ischemic stroke is mediated largely by immune cells, including T- and B-cells, microglia, macrophages, and neutrophils, that invade the brain following BBB rupture (Li et al., 2021c; Jiang et al., 2022). An exaggerated immune response further contributes to cell death and cognitive dysfunction after stroke (Doyle et al., 2015; Jiang et al., 2022). Microglia are quickly activated to the inflammatory M1 phenotype and remain activated for at least 14 days, releasing many factors that exacerbate the inflammatory environment of the brain and the degradation of the BBB (Hu et al., 2012; Jiang et al., 2022). Glutamate excitotoxicity is elicited following the initial cascade of cell-mediated inflammation in the post-stroke brain, leading to more cell death via oxidative stress and electrolyte disturbances (Jiang et al., 2022).

Hemorrhagic stroke, on the other hand, is caused by intracerebral hemorrhage, resulting in hematoma and local neuron loss (Hilkens et al., 2024). Much of the primary damage is caused by the expansion of the hematoma either in the cerebral parenchyma or the subarachnoid space (Maida et al., 2024). The mechanisms of molecular injury in hemorrhagic stroke resemble those in ischemic stroke, including glial activation, neuroinflammation, oxidative stress, and BBB disruption (Maida et al., 2024); however, a key difference may be the pathogenic presence of iron, hemoglobin, and carbonic anhydrase from the extravasated blood (Zhang R. et al., 2020).

#### 3.5.1 MSC preclinical trials in stroke

Early preclinical studies showed that MSCs were safe and effective in treating stroke. One of the first studies to use MSCs in a preclinical model of stroke demonstrated that BM-MSCs injected intravenously improved outcomes over controls. Interestingly, the authors also observed that MSCs migrated to the brain but remained mostly undifferentiated (Chen et al., 2001). The authors hypothesized this could be due to the production of cytokines and trophic factors which boost endogenous mechanisms of regeneration, which is consistent with a paracrine signaling mechanism of action (Chen et al., 2001). Shortly following this study, Kang et al. used human AD-MSCs induced toward a neural cell morphology in rat MCAO stroke models and observed improved outcomes. These findings were enhanced when the MSCs were transduced to express BDNF, demonstrating the viability of MSCs as delivery vehicle for trophic factor secretion in neurologic disease (Kang et al., 2003). A long-term follow-up study in a rat model of stroke reported that MSCs promote neurologic recovery as long as 1 year post-MCAO (Shen et al., 2007).

For stroke in particular, the timing of treatment is of great concern. Traditional thrombolytic and clot retrieval therapies must be administered in under 4.5 h which is often unattainable in clinical practice (Cunningham et al., 2018). A preclinical study by Toyoshima et al. demonstrated that the optimal timing of allogenic MSC administration for MCAO models of stroke was 24 h (Toyoshima et al., 2015), though other studies suggest that MSCs remain beneficial as long as 3 days after MCAO (Cunningham et al., 2018). Another study reported that two infusions of MSCs at 8 and 24 h post stroke was more efficacious than a single infusion at 24 h, which supports a multiple-dose treatment strategy (Kranz et al., 2010). While the optimal time and dosing strategy is still up for debate, it is clear that MSC infusion following stroke has a longer effective time frame than conventional therapies while still providing significant neurological recovery.

MSC exosomes have also shown therapeutic benefit in preclinical models of stroke. The first preclinical study to investigate exosomes in the treatment of stroke was performed by Xin et al. (2013a). The investigators treated MCAO rats with 100 μg of BM-MSC-derived exosomes 24 h post-MCAO. They found that rats treated with BM-MSC-exosomes showed significant improvement in motor function and improved neurogenesis, angiogenesis and enhanced neural remodeling (Xin et al., 2013a). Doeppner et al. confirmed these findings by comparing human BM-MSC-derived exosomes to human BM-MSCs in a mouse MCAO model. The BM-MSC exosomes exerted a similar effect to the MSCs *in vivo*, making this one of the first studies to demonstrate the efficacy of *ex vivo* harvested MSC-derived exosomes alone in the treatment of ischemic stroke (Doeppner et al., 2015). Another combinatorial strategy found that BMSC exosomes combined with rosuvastatin in rats decreased infarcted lesion size and reduced glial scarring (Safakheil and Safakheil, 2020).

#### 3.5.2 MSC clinical trials in stroke

The earliest clinical trial investigating the use of MSCs for the treatment of ischemic stroke was reported in 2005. In this study 5 patients treated with autologous BM-MSCs demonstrated improvements on the Rankin score and Barthel Index (BI) with no treatment-related adverse events (Bang et al., 2005). A 5-year follow-up trial involving 52 patients found that the treatment group had lower overall mortality compared to the control group, while no differences in comorbidities between groups were observed during the follow-up period (Lee et al., 2010).

Another notable clinical trial evaluating autologous BM-MSCs administered to 12 stroke patients between 36 and 133 days post-stroke reported over 20% lesion volume reduction (Honmou et al., 2011). Concurrent trials confirmed the safety of BM-MSCs for use in treating stroke patients. Bhasin et al. reported no treatment-related adverse events but only slight improvements (Bhasin et al., 2011); however, a second trial showed no adverse reactions with a statistically significant improvement on the modified Barthel Index (mBI) in the treated group (Bhasin et al., 2013).

Recent clinical trials have explored alternative administration routes and innovative stem cell formulations. A notable phase 1/2 dose-escalation trial (NCT01287936) administered BM-MSCs transfected with the Notch-1 gene via stereotactic implantation in chronic ischemic stroke patients. Transfection with the *Notch-1* plasmid is transient, and transfected cells do not survive overlong *in vivo*, minimizing risk of unanticipated side effects from *Notch-1* expression. Treated patients experienced clinically meaningful improvements (Steinberg et al., 2016), thus confirming the feasibility of transiently manipulated MSCs for human stroke treatment. Another phase 2 clinical trial (NCT04811651) investigating the efficacy of allogenic hUC-MSCs against placebo in 156 patients with acute stroke has been recently completed, but to our knowledge results have not yet been published.

The first phase 3 clinical trial (NCT01716481) of MSCs in stroke patients was conducted in South Korea from 2012 through 2017. Autologous BM-MSCs preconditioned with post-stroke autologous serum were administered intravenously in 54 patients with acute ischemic stroke. Despite a positive safety profile, no significant therapeutic efficacy was demonstrated (Chung et al., 2021). Similarly, a sequence of clinical trials investigating the allogenic bone marrow-derived mesenchymal progenitor cell product MultiStem HLCM501 (allogenic BM-multipotent progenitor cells) have shown positive safety data but failed to yield significant clinical results (Hess et al., 2017; Houkin et al., 2024). These trials have culminated in a phase 3 clinical trial (NCT03545607), but the status of this trial remains unknown.

Emerging trials have targeted different forms of stroke. In 2022 Baak et al. published results from a phase 1/2 study (NCT03356821) investigating intranasal allogenic BM-MSC administration for perinatal arterial ischemic stroke. Results showed promising safety but no significant clinical improvements (Baak et al., 2022). Additionally, a small phase 1 trial (NCT03371329) in patients with acute intracerebral hemorrhage demonstrated MSC treatment feasibility along with increased anti-inflammatory cytokines CD40L, IL-1RA, and IL-10 up to 3 days after treatment, suggesting that MSCs may attenuate acute inflammation in hemorrhagic stroke (Durand et al., 2024).

There are a number of upcoming and in-progress clinical trials involving MSCs for stroke treatment. A phase 2/3 study (NCT06129175) will investigate allogenic hUC-MSCs in 80 patients with acute stroke. A phase 1/2 study (NCT06997939) investigating autologous BM-MSCs was recently registered. This study plans to recruit 12 patients with ischemic stroke and measure the efficacy of Ommaya drug reservoir administration versus internal carotid artery transplantation. Notably, two upcoming clinical trials plan to study the therapeutic efficacy of MSC-exosomes for stroke. The first (NCT05158101) will study exosomes derived from allogenic hUC-MSCs. 15 stroke patients will be recruited, and 2 doses of approximately 800 billion exosomes will be administered intranasally. This trial is expected to conclude in 2026. Another phase 1 clinical trial (NCT06995625) will study allogenic WJ-MSC exosomes in the treatment of ischemic stroke. This study will treat 18 patients in a dose escalation design to determine the maximum tolerated dose. This study is expected to be completed in 2027. Table 5 provides a summary of clinical trials investigating MSCs for treatment of stroke.

**TABLE 5:** Clinical trials investigating MSC-based therapies for stroke.

Trial ID, status, phase	Study design	# PPTS	Cell-type and route of administration	Intervention	Results	Citation
NA, Completed, Phase ½	Randomized, controlled, parallel assignment	30 patients w/MCA ischemic stroke	Autologous BM-MSCs, Intravenous	1 infusion of 1.0 * 10^8^ cells	Barthel index and modified Rankin Scale (mRS) scores improved in MSC group.	(Bang et al., 2005)
NA, Completed, -	Open label, randomized, controlled	85 patients w/severe MCA ischemic stroke	Autologous BM-MSCs, Intravenous	2 infusions of 2.0 *10^7^ cells, at two week intervals	No significant side effects observed during follow up period. Modest decrease in mRS in treatment group	(Lee et al., 2010)
NA, Completed, Phase 1	Open label, single arm	12 patients w/ischemic stroke < 6 months	Autologous BM-MSCs, Intravenous	1 infusion of 0.6–1.6 * 10^8^ cells	No significant side effects observed. > 20% reduced lesion volume in treatment group at 1 week	(Honmou et al., 2011)
NA, Completed, -	Non-randomized, controlled, parallel assignment	12 patients w/chronic stroke (3 months – 1 year)	Autologous BM-MSCs, Intravenous	1 infusion of 50–60 * 10^6^ cells + 8 weeks of physiotherapy.	No treatment-related adverse events. Fugl-Meyer and mBI increased slightly in treatment group	(Bhasin et al., 2011)
NA, Completed, -	Non-randomized, open label, controlled, parallel assignment	40 patients w/stroke	Autologous BM-MSCs, Intravenous	50–60 *10^6^ cells.	Modest improvement in mBI only over controls.	(Bhasin et al., 2013)
NCT01287936, Completed, Phase 1/2	Open label, randomized, parallel assignment	18 patients w/chronic stable ischemic stroke	SB623 ^®^ (Allogenic BM-MSCs transfected with Notch-1 gene), Stereotactic Implantation	1 implantation of 2.5, 5.0, or 10.0 * 10^6^ cells	No Adverse events related to treatment. Clinically meaningful improvements in the ESS, NIHSS, F-M total score, and F-M motor function scores were observed.	(Steinberg et al., 2016)
NCT01436487, Completed, Phase 2	Randomized, double blind, placebo-controlled, parallel assignment, dose escalation	134 patients w/acute cortical cerebral ischemic stroke (< 36 h)	MultiStem HLCM051 ^®^ (allogenic BM-multipotent progenitor cells), Intravenous	Group 1: 4.0 * 10^8^ cells. Group 2: 1.2 * 10^9^ cells. Group 3: 1.2 * 10^9^ cells.	No does limiting adverse events observed. No significant difference between groups in stroke recovery at day 90	(Hess et al., 2017)
NCT01297413, Completed, Phase 1/2	Open label, single arm	38 patients w/ischemic stroke (> 6 months)	Allogenic BM-MSCs, Intravenous	1 infusion of 0.5–1.5 * 10^6^ cells	No serious adverse events related to treatment. Significant improvements in all behavioral endpoints	(Levy et al., 2019)
NCT00875654, Completed, Phase 2	Open label, randomized, controlled, parallel assignment	31 patients w/acute R or L carotid ischemic stroke (< 14 days)	Autologous BM-MSCs, Intravenous	1 infusion either 1.0 or 3.0 * 10^8^ cells + rehabilitation	No treatment emergent adverse events. No changes in global improvement scales like NIHSS, Barthel Index or mRS. Some improvements in FM motor score and task related fMRI activity.	(Jaillard et al., 2020)
NCT01716481, Completed, Phase 3	Randomized, open label, placebo controlled, parallel assignment study	54 patients w/MCA ischemic stroke (< 90 days)	Autologous BM-MSCs, Intravenous	1 infusion of 1.0 * 10^6^ cells/kg	Autologous MSC treatment is safe in patients with chronic stroke, but did not improve outcomes	(Chung et al., 2021)
NCT03356821, Completed, Phase 1/2	Open label, single arm	10 infants w/perinatal arterial ischemic stroke (PAIS) (< 1 week)	Allogenic BM-MSCs, Intranasal	1 infusion of 50 * 10^6^ cells	No serious treatment-related adverse events were observed	(Baak et al., 2022)
NCT02961504, Completed, Phase 2/3	Randomized, double-blind, placebo controlled, parallel assignment	206 patients w/acute ischemic stroke (18–36 h before administration)	MultiStem HLCM051 ^®^ (allogenic BM-multipotent progenitor cells), Intravenous	1 infusion of 1.2 billion cells 18–36 h after stroke	Trial failed to meet primary efficacy endpoint: No difference in outcome at 90 days compared to control.	(Houkin et al., 2024)
NCT03371329, Completed, Phase 1	Non-randomized, open label, sequential assignment, dose escalation	9 patients w/acute ICH	Allogenic BM-MSCs, Intravenous	1 infusion of 0.5, 1.0, or 2.0 * 10^6 cells/kg	1 possible treatment-related adverse event of fever reported. Increases in anti-inflammatory cytokines CD40L, IL-1 receptor antagonist, and IL-10 were observed	(Durand et al., 2024)
NCT04811651, Completed, Phase 2	Randomized, double blind, placebo controlled, parallel assignment	156 patients w/ischemic stroke < 6 months	Allogenic hUC-MSCs, Intravenous	1 infusion of 1.0 * 10^8^ cells	Not yet published	N/A
NCT05008588, Unknown, Phase 1/2	Open label, randomized, controlled	15 patients w/acute ischemic stroke	Allogenic hUC-MSCS, Intranasal + Intraparenchymal	Group 1: intraparenchymal transplantation of 20 * 10^6^ cells + 3 intranasal administrations of conditioned medium. Group 2: intraparenchymal transplantation of 20 * 10^6^ cells.	Not yet published	N/A
NCT03545607, Unknown, Phase 3	Randomized, double-blind, placebo controlled, parallel assignment	300 patients w/ischemic stroke (18–36 h)	MultiStem HLCM051 ^®^ (allogenic BM-multipotent progenitor cells), Intravenous	1 infusion of 1.2 billion cells 18–36 h after stroke	Not yet published	N/A
NCT06752720, Recruiting, Phase 2	Open label, single arm	8 patients w/chronic ischemic stroke (> 6 months)	Autologous BM-MSCs, Intracerebral	1 injection of 4.0 * 10^7^ cells	–	N/A
NCT05158101, Recruiting, Phase 1	Open label, single arm	15 patients w/stroke	AlloEx ^®^ (Allogenic hUC-MSC-derived exosomes), Intranasal	2 doses of ∼800 billion exosomes, administered on 2 consecutive days	–	N/A
NCT04097652, Recruiting, Phase 1	Open label, single arm, dose escalation study	9 patients w/ischemic stroke within 48–168 h before administration	Allogenic hUC-MSCs, Intravenous	Group 1: low dose. Group 2: Medium dose. Group 3: High dose. Specific dosage unspecified	–	N/A
NCT06129175, Recruiting, Phase 2/3	Randomized, double blind, placebo controlled, parallel assignment	80 patients w/ischemic stroke < 4 weeks	Neuroncell-EX ^®^ (Allogenic hUC-MSCs), Intravenous	2 infusions of 2.0 * 10^6^ cells/kg. Administered on days 1 and 14	–	N/A
NCT06518902, Not Yet Recruiting, Phase 1	Open label, single arm	18 patients w/acute ischemic stroke (< 1 month)	Allogenic hUC-MSCs, Unspecificed	1 injection or 3 injection, once a week for 3 weeks. Dosage unspecified	–	N/A
NCT06997939, Not Yet Recruiting, Phase 1/2	Randomized, double blind, parallel assignment	12 patients w/ischemic stroke 3–12 months	Autologous BM-MSCs, Intracerebral (Ommaya reservoir) or Intraarterial (Internal Carotid Artery)	Ommaya reservoir group: 2.0 * 10^7^ cells administered via Ommaya reservoir. Low-dose intra-arterial group: 2.5 *10^6^ cells via internal carotid artery. High-dose intra-arterial group: 1.0 * 10^7^ cells via internal carotid artery	–	N/A
NCT07084012, Not Yet Recruiting, Phase 2	Randomized, double blind, parallel assignment, placebo controlled	60 patients w/acute ischemic stroke (< 72 h)	Allogenic hUC-MSCs, Intravenous	Group 1: 2.0 * 10^8^ cells on day 0, then placebo on days 7 and 14. Group 2: 1.0 * 10^8^ cells on day 0, 7, and 14. Group 3: 3 Placebo doses on day 0, 7, and 14.	–	N/A
NCT06995625, Not Yet Recruiting, Phase 1	Open label, single arm, dose escalation study	18 patients w/ischemic stroke (< 5 days)	Allogenic WJ-MSC-derived exosomes, Intravenous	Group 1: 4.8 * 10^10^ exosomes. Group 2: 9.6 * 10^10^ exosomes. Group 3: 19.2 * 10^10^ exosomes. Exosomes administered once a day for 5 days	–	N/A

Data in this table were gathered using clinicaltrials.gov (Clinicaltrials.gov, 2025) or through consulting the corresponding reference listed in the table. References are provided only if the clinical trial results have been published. Note: clinicaltrials.gov is not always up to date. Presented data reflect data in corresponding publications where available. MCA, Middle Cerebral Artery; ICH, Intracerebral Hemorrhage; BI, Barthel Index; mBI, Modified Barthel Index; FM, Fugl-Meyer; ESS, European Stroke Scale; NIHSS, National Institute of Health Stroke Scale. Efforts were made to include completed trials as well as those in the “active, not recruiting,” “recruiting,” and “not yet recruiting” phases. Clinical trials with unknown status are included when appropriate.

### 3.6 Spinal cord injury

Spinal cord injury results from traumatic mechanical damage to the spinal cord, causing motor and sensory deficits as well as autonomic dysfunction. It is characterized pathologically by intense inflammation, vasospasms and ischemia, excitotoxicity, oxidative stress, breakdown of the blood spinal cord barrier, and neuronal apoptosis (Anjum et al., 2020). SCI occurs in two phases: the primary injury, characterized by the acute and sudden trauma to the spine, and secondary injury, which is comprised of long-term neuroinflammation and glial scarring (Hu et al., 2023). Primary injury-related vasospasm and vasogenic edema occur rapidly, resulting in local tissue dysfunction and necrosis. Neuronal death leads to glutamate excitotoxicity, perpetuating inflammatory and apoptotic cascades. Microglia and astrocytes, normally responsible for maintaining homeostatic balance in neural tissue, become pro-inflammatory in the presence of inflammatory cytokines released by invading neutrophils. Cytotoxic T-cells have also been observed at the site of SCI where they may exacerbate inflammation and cytotoxicity (Anjum et al., 2020; Hu et al., 2023). Furthermore, the prolonged inflammatory microenvironment eventually leads to axonal degeneration and demyelination, followed by the formation of a fibrotic scar which poses a physical barrier to axonal regeneration (Anjum et al., 2020; Hu et al., 2023).

Injury severity and level of disability in SCI is typically measured using the American Spinal Injury Association (ASIA) Impairment Scale (AIS) scoring system, which scores patients with SCI on a scale from A to E. A represents complete injury of the spinal cord with no sensory or motor function below the injury. B, C, and D represent varying levels of incomplete injury, with B describing preserved sensory function but no motor function below the injury, and C and D describing weakened motor function. An ASIA score of E represents no motor or sensory deficit (McDonald and Sadowsky, 2002).

#### 3.6.1 MSC preclinical trials in SCI

*In vitro* and animal models have served as vital tools for studying the beneficial effects of MSCs in treating SCI since the early 2000s. The first study to demonstrate SCI-specific safety and feasibility of MSCs was conducted by Chopp et al. (2000) using a rat model SCI induced via weight drop method. The MSC treatment showed significant functional improvement in the injured rats, and the authors theorized that the MSCs could be acting through a paracrine signaling mechanism (Chopp et al., 2000), a theory that would be explored as the field evolved (Liu et al., 2019).

Since the characterization of their therapeutic benefit in SCI, another promising avenue of research in preclinical studies is the adjunctive use of hydrogels and collagen scaffolds alongside MSC transplantation, which can improve MSC survival and efficacy (Lv et al., 2021; Pang et al., 2021; Silva et al., 2021). In an experiment by Papa et al. a biomimetic hydrogel scaffold was loaded with hUCB-MSCs and grafted into a mouse model of SCI. The engrafted mice showed improved cytoskeletal architecture preservation, increased neuronal survival, and M2 macrophage proliferation, likely through the release chemokine ligand 2 (CCL2) (Papa et al., 2018). The same result was observed in canine models of SCI which showed axonal regeneration following hUC-MSC impregnated collagen scaffolds surgically implanted into the thoracic spinal cord of canines (Li et al., 2017).

In addition to scaffolds, the combination of MSCs with pharmaceuticals and/or genetically altered MSCs has emerged as a promising therapeutic strategy. One such study by Lee et al. combined AD-MSCs with chondroitinase ABC (chABC), an enzyme capable of breaking down chondroitin sulfate proteoglycan (CSPG) glial scars, which improved SCI functional outcomes in canines (Lee et al., 2015). AD-MSCs engineered to express chABC also yielded significant functional improvements in rat models of SCI (Lu et al., 2020). Furthermore, hUC-MSCs transduced with an IL-10-producing-vector improved functional outcomes, reduced lesion size, and improved axonal regeneration in mice (Gao et al., 2022). The same was true of MSCs engineered to overexpress IL-13 (Dooley et al., 2016).

#### 3.6.2 MSC clinical trials in SCI

There have been many attempts to translate the results of preclinical trials of MSCs in SCI to human trials with mixed results. Early treatment approaches with MSCs primarily employed BMMNCs and yielded promising results (Chernykh et al., 2007; Yoon et al., 2007; Kumar et al., 2009). Subsequent MSC-specific clinical trials demonstrated consistent safety data across a range of MSC sources. One phase 1 trial (NCT01274975) used autologous AD-MSCs in 8 patients with SCI and observed no adverse events or tumorigenicity (Ra et al., 2011). Another phase 1 trial (NCT01325103) in 14 subjects investigated BM-MSCs demonstrated improvement in AIS scores and urologic function (Mendonça et al., 2014).

More recent clinical trials have supported the safety and modest efficacy of MSCs in SCI (Oh et al., 2016; Satti et al., 2016; Yang et al., 2021). A phase 1 study (NCT02482194) in 9 patients administered BM-MSCs through intrathecal injection showed no treatment-related adverse events (Satti et al., 2016). A phase 1/2 clinical trial (NCT02481440) using allogenic hUC-MSCs in 102 patients with SCI at various locations also showed no serious treatment-related adverse events and even demonstrated improvements in ASIA and IANR-SCIFRS scores (Yang et al., 2021). Most notable, perhaps, is the CELLTOP trial, a phase 1 clinical trial (NCT0330856) in ten patients treated with autologous AD-MSCs. A preliminary report on the progress of the first patient in the study, who suffered an AIS grade A C3-C4 SCI, detailed remarkable clinical improvement (Bydon et al., 2020), which generated considerable excitement about the potential of AD-MSCs for treating SCI. The results from the full phase 1 trial (NCT03308565) described improvement in AIS score in 7 of the 10 participants (Bydon et al., 2024).

A pilot study in 10 patients with ASIA grade A or B demonstrated that intramedullary injection of BM-MSCs during laminectomy followed by 2 subsequent intrathecal injections of BM-MSCs improved long-term motor function in 3 patients and reduced glial scar formation in 2 patients (Park et al., 2012), a phenomenon consistent with preclinical studies (Anjum et al., 2020). A phase 2/3 clinical trial (NCT01676441) was initiated based on the results of this pilot study. The same procedure of intramedullary injection following laminectomy was employed, but only one injection was performed due to restrictions around multiple administrations for phase 3 trials. The trial ultimately failed to meet its efficacy endpoint, possibly due to the reduction of treatment rounds (Oh et al., 2016).

Studies that followed have continued to demonstrate the safety and potential of MSCs in treating SCI. A phase 1/2 study of allogenic WJ-MSCs in 10 patients (NCT03003364) with thoracic SCI showed significant improvement in pinprick sensation below the level of injury and a reduction in bladder symptoms (Albu et al., 2021). Similarly, a phase 1/2 repeat dose study (NCT02981576) investigating the efficacy of BM-MSCs vs. AD-MSCs intrathecal injections in 14 patients showed improvements in 4 patients, and one patient even regained the ability to walk (Jamali et al., 2024). Notably, a phase 1 clinical trial (NCT02352077) provided first in-human evidence for the use of collagen scaffold (NeuroRegen) use alongside MSCs. In this study 8 patients with SCI received surgical implantation of a scaffold loaded with hUC-MSCs. The majority of patients experienced an expansion of sensation level and motor-evoked potential (MEP) responsive area, but no overall improvement was seen in ASIA scores (Zhao et al., 2017). Another clinical trial using NeuroRegen scaffolds loaded with BMMNCs in combination with a 6-month course of standardized rehabilitation showed sensory improvement, but no motor improvement. A follow-up study (NCT02688049) investigating the NeuroRegen scaffold in combination with either MSCs or neural stem cells has been initiated, but the status of this trial remains unknown.

There are three notable upcoming clinical trials studying MSCs for SCI. A phase 1 trial (NCT05018793) will recruit 15 patients with SCI and compare the efficacy of autologous AD-MSCs against allogenic hUC-MSCs. The CELLTOP part II trial (NCT04520373) will enroll 40 patients with SCI and compare the efficacy of autologous AD-MSCs to best medical management, alongside occupational and physical therapy. Finally, a phase 1/2 trial (NCT06981338) will combine allogenic WJ-MSCs with transcutaneous spinal cord stimulation and neurorehabilitation for the first time in humans. Table 6 describes the current state of clinical trials involving MSCs for treatment of SCI.

**TABLE 6:** Clinical trials investigating MSC-based therapies for SCI.

Trial ID, status, phase	Study design	# PPTS	Cell-type and route of administration	Intervention	Results	Citation
NA, Completed, Phase 1	Open label, single arm	6 patients w/complete SCI	Autologous bone marrow stem cells, Intralesional	1.1 * 10^6^ cells	Treatment found to be safe overall. 4 patients experienced improvement in AIS grade	(Park et al., 2005)
NA, Completed, Phase 1/2	Non-random, open-label, controlled, observer blinded	53 patients w/SCI, AIS grade A	BMMNCs + GM-CSF, Laminectomy Followed by Injection	1 injection of 2.0 * 10^8^ cells	Improvement in AIS score in 30.4% of patients in the acute and subacute SCI groups. No improvement in chronic SCI group.	(Yoon et al., 2007)
NA, Completed, Phase 1	Randomized, controlled.	36 patients w/chronic SCI	Autologous bone marrow stem cells, Surgical Implantation + Intravenous	Dosage unspecified	Treatment well tolerated. 12 of 18 patients in the treatment group experienced improvement in motor and sensory function	(Chernykh et al., 2007)
NA, Completed, Phase 1/2	Non-randomized, open label, uncontrolled	297 patients w/traumatic paraplegia (*n* = 215), traumatic quadriplegia (n = 49) and non-traumatic spinal cord myelopathy (*n* = 33)	Autologous BMMNCs, Intrathecal	Dosage unspecified	32.6% of patients experienced sensory and motor improvements after treatment	(Kumar et al., 2009)
NCT01274975, Completed, Phase 1	Open label, single arm	8 patients w/SCI AIS grade A, B, or C	AstroStem ^®^ (Autologous AD-MSCs), Intravenous	1 infusion of 4.0 * 10^8^ cells	No serious adverse events occurred	(Ra et al., 2011)
NA, Completed, Phase 1	Open label, single arm	10 patients w/SCI	Autologous BM-MSCs, Laminectomy followed by Injection	8.0 * 10^6^ MSCs intramedullary and 4.0 * 10^7^ cells intradural, followed by 5.0 * 10^7^ cells via lumbar tapping at weeks 4 and 8	Improved long-term motor function in 3 patients and reduced glial scar formation in 2 patients	(Park et al., 2012)
NCT01325103, Completed, Phase 1	Open label, single arm	14 patients w/thoracic or lumbar SCI AIS grade A	Autologous BM-MSCs, Intralesional	5.0 * 10^6^ cells/cm^3^ lesion volume	Treatment safe and well-tolerated. 8 patients had lower limb functional improvement. 7 patients improved on AIS score. 9 patients had improved urological function.	(Mendonça et al., 2014)
NCT02482194, Completed, Phase 1	Open label, single arm	9 Patients w/thoracic SCI; AIS grade A	Autologous BM-MSCs, Intrathecal	2 or 3 doses of 1.2 * 10^6^ cells/kg	No treatment related adverse events observed.	(Satti et al., 2016)
NCT02352077, Completed, Phase 1	Open label, single group assignment study	8 patients with SCI at C5-T12; AIS grade A	NeuroRegen Scaffold ^®^ loaded w/hUC-MSCs, Surgical Implantation	Scar tissue resection followed by surgical implantation of NeuroRegen Scaffold loaded with 4.0 * 10^7^ cells hUC-MSCs.	62.5% of Patients experienced expansion of sensation. 3 patients showed increased finger flexibility. 87.5% saw expansion of Motor-evoked potential-responsive area. Autonomic function was also detected below level of injury.	(Zhao et al., 2017)
NCT02510365, Completed, Phase 1	Open Label, single arm	7 patients w/SCI AIS grade A between T1-T12	NeuroRegen Scaffold ^®^ loaded w/BMMNCs, Laminectomy Followed by Implantation	Surgical implantation of NeuroRegen Scaffold loaded with ∼1.0 *10^9 cells	Partial sensory recovery observed. No motor recovery observed.	(Chen W. et al., 2020)
NCT03003364, Completed, Phase 1/2	Randomized, double-blind, crossover, placebo-controlled	10 patients w/chronic completed SCI at T2-T11; ASIA grade A	Allogenic WJ hUC-MSCs, Intrathecal	1 injection of 10.0 * 10^6^ cells	No treatment related adverse events. Significant improvement in pinprick sensation below injury. Reduction in bladder symptoms.	(Albu et al., 2021)
NCT02481440, Completed, Phase 1/2	Open label, single arm	102 patients w/SCI C1—L1	Allogenic hUC-MSCs, Intrathecal	4 injections of 1.0 * 10^6^ cells/kg	No serious adverse events. Improvements in ASIA and IANR-SCIFRS score. Reduced muscle spasticity	(Yang et al., 2021)
NCT02981576, Completed, Phase 1/2	Randomized, open label, parallel assignment	14 patients w/SCI AIS grade A, B, or incomplete grade C	Autologous BM-MSCs vs. autologous AD-MSCs, Intrathecal	3 injections of 1.0 * 10^8^ cells (either BM-MSCs or AD-MSCs)	No serious treatment emergent adverse events in either group. AD-MSC group experienced better motor and sensory recovery	(Jamali et al., 2024)
NCT03308565, Completed, Phase 1	Open label, single arm	10 patients w/SCI < 1 year; AIS grade A or B	Autologous AD-MSCs, Intrathecal	1 injection of 1.0 * 10^8^ cells	No Serious Adverse Events. 7 patients demonstrated improvement in AIS score	(Bydon et al., 2024)
NCT02688049, Unknown Status, Phase 1/2	Randomized, double blind, parallel assignment	30 patients w/SCI ASIA grade A between C5-T12	NeuroRegen Scaffold ^®^ loaded w/MSCS or NSCs, Intraspinal	Implantation of NeuroRegen scaffold loaded with either 1.0 * 10^7^ MSCs, or 1.0 * 10^7^ NSCs	Not yet published	N/A
NCT01676441, Terminated, Phase 2/3	Open label, single arm	20 patients w/chronic cervical SCI; AIS grade B	Autologous BM-MSCs, Intramedullary + Intrathecal	1.6 * 10^7^ cells intramedullary + 3.2 * 10^7^ cells intrathecally	No adverse effects of treatment. Failure to meet primary efficacy endpoint. Only 2 PPTs showed neurological improvement	(Oh et al., 2016)
NCT05018793, Recruiting, Phase 1	Open label, single arm	15 patients with SCI	Autologous AD-MSCs vs. Allogenic hUC-MSCs, Intrathecal	1 injection of 1.0 * 10^8^ cells	–	N/A
NCT04520373, Recruiting, Phase 2	Randomized, open-label, controlled, crossover study	40 patients w/SCI; ASIA grade A or B	Autologous AD-MSCs, Intrathecal	1 injection. Dose unspecified	–	N/A
NCT06981338, Not Yet Recruiting, Phase 1/2	Open label, single arm	10 patients w/chronic SCI C1-T12; AIS grade A-C	Allogenic WJ-MSCs, Intrathecal	3 Injections of 30 * 10^6^ cells + transcutaneous spinal cord stimulation assisted neurorehabilitation	–	N/A

Data in this table were gathered using clinicaltrials.gov (Clinicaltrials.gov, 2025) or through consulting the corresponding reference listed in the table. References are provided only if the clinical trial results have been published. Note: clinicaltrials.gov is not always up to date. Presented data reflect corresponding publications where available. GM-CSF, Granulocyte macrophage colony stimulating factor; ASIA, American Spinal Injury Association; AIS, ASIA Impairment Scale; IANR-SCIFRS, International Association of Neurorestoratology SCI Functional Rating Scale. Efforts were made to include completed trials as well as those in the “active, not recruiting,” “recruiting,” and “not yet recruiting” phases. Clinical trials with “unknown” or “terminated” status are included when appropriate.

### 3.7 Traumatic brain injury

Traumatic Brain Injury is a major preventable cause of death and disability in adults under 40 and affects nearly 50 million people worldwide every year (Khellaf et al., 2019). Symptoms and sequalae vary widely depending on the severity and nature of the injury; TBI is closely associated with chronic traumatic encephalopathy (CTE), a neurodegenerative condition associated with the deposition of phosphorylated tau in perivascular regions in the brain. The deposits have been linked with widespread cortical atrophy and structural changes in the ventricles, corpus callosum, cavum septum pellucidum, and cerebellum (McKee et al., 2023), resulting in altered brain physiology and cognitive deficits (McKee et al., 2009). TBI and CTE may also cause psychiatric symptoms, including depression, anxiety, disinhibition, post-traumatic stress disorder (PTSD), and suicidality (Howlett et al., 2022).

The initial mechanical injury in TBI results in instantaneous cell death and diffuse axonal injury. Following the initial injury there is a phase of acute inflammation characterized by microglial and astrocyte activation, increase in inflammatory cytokines like TNF-α, IL-1β, and IL-6, BBB damage, oxidative stress, and increase in levels of matrix metalloproteinases (MMPs) leading to further neuronal damage and BBB dysfunction. This acute phase of inflammation precipitates a chronic state of inflammation that can lead to neurodegeneration (Roberts et al., 1991; McKee et al., 2009; Blennow et al., 2016; Kaniuk et al., 2025).

Severity of TBI is commonly assessed using the Glasgow coma scale (GCS), a clinical tool which measures an individual’s level of consciousness. The GCS contains assays for visual, verbal, and motor function, and is thus able to summarize many multi-faceted symptoms of TBI (Bonilla and Zurita, 2021).

#### 3.7.1 MSC Preclinical studies in TBI

While other types of stem cells have shown benefit in treating TBI, the first preclinical trial of specifically BM-MSCs in treating TBI was conducted by (Mahmood et al. (2001). Despite the positive motor function results of the study, the mechanism of therapeutic effect was not fully explored (Mahmood et al., 2001). Subsequent work has suggested that MSCs primarily operate through paracrine signaling upon administration; however, there is conflicting evidence from other preclinical studies suggesting that intravenous injection of MSCs may not be as effective as other routes of administration (Harting et al., 2009). MSCs may also target the pathological build-up of tau post TBI. Tau accumulation in the TBI brain follows a unique pattern whereby it selectively deposits in perivascular regions within the sulci of the brain (McKee et al., 2009). A recent study investigating MSC-exosomes in a mouse model of TBI provided evidence that MSC-exosomes could reduce tau burden after TBI. The TBI mice were administered human BM-MSC-derived-exosomes loaded with anti-*cis* phosphorylated tau (p-tau) antibodies. Lower levels of *cis* p-tau, and cleaved caspase-3 as well as higher levels of p-PI3K were observed, which correlated with greater functional recovery (Amini et al., 2023). While MSCs have been shown to improve clearance of Aβ in AD models (Lee et al., 2009; Qin et al., 2022), Aβ deposition, while frequent in TBI, is less strongly associated with TBI (McKee et al., 2009), thus Aβ clearance may not be a primary mechanism by which MSCs exert their therapeutic effect.

#### 3.7.2 MSC clinical trials in TBI

The first major clinical trial investigating the safety and efficacy of BMMNCs for treating TBI was conducted by Cox et al. in pediatric patients (NCT00254722). 10 pediatric patients from 5-14 years old were administered autologous BMMNCs intravenously within 48 h of TBI. The treatment was well-tolerated overall with no detectable treatment-related toxicities (Cox et al., 2011). A retrospective cohort study performed with the data from this trial also showed that autologous BMMNC treatment following TBI reduced the intensity of medical therapy in the intensive care unit compared to controls (Liao et al., 2015). Following this study, Cox et al. conducted a placebo-controlled clinical trial (NCT01575470) using autologous BMMNCs administered in adults within 48 h after TBI. Autologous BMMNC treatment was safe and even reduced levels of inflammatory cytokines relative to controls (Cox et al., 2017). BMMNCs may also improve outcomes in chronic TBI. Sharma et al. reported the results of a pilot study on autologous BMMNCs administered intrathecally in 14 patients with chronic TBI. 14 patients were recruited and administered BMMNCs intrathecally alongside a course of neurorehabilitation therapy. Multiple participants experienced improvements in various domains of motor function and activities of daily living (Sharma et al., 2015). Though this study yielded promising results, larger, controlled studies will be needed to fully characterize the activity of BMMNCs in chronic TBI.

In 2013, Wang et al. reported the results of controlled clinical trial in 40 patients with sequelae of TBI between 1 and 11 years after injury. Patients were administered allogenic hUC-MSCs intrathecally. Fugl-Meyer Assessment (FMA) and Functional Independence Measure (FIM) scores were obtained at baseline and after 6 months of follow-up. Treated participants demonstrated clinical improvement in motor function and independence as measured by the FMA and FIM respectively, whereas controls exhibited no clinical improvement (Wang et al., 2013). This study provided strong evidence that hUC-MSC therapy is beneficial in treating chronic TBI.

One recent phase 2 clinical trial (NCT02416492) investigated the efficacy of modified MSCs in patients with TBI. 46 patients in the treatment group underwent stereotactic implantation with allogenic BM-MSCs transiently modified with a *Notch-1* plasmid, which inhibited their ability to differentiate into other cell types. Only 1 patient in the treatment group experienced a serious treatment emergent adverse event possibly related to the cell treatment. Overall, the treatment was shown to significantly improve motor function compared to controls as measured by the Fugl-Meyer motor score (Kawabori et al., 2021). The results of another phase 2 trial using autologous BMMNCs were recently reported by Cox et al. (2024) (NCT01851083). This trial expanded on a previous phase 1 trial (Cox et al., 2011) by implementing a randomized, double blind, placebo-controlled design. 47 pediatric patients with acute TBI (< 48 h) and GCS score 3-8 were recruited. Patients treated with autologous BMMNCs experienced shorter time on the ventilator and in the intensive care unit (Cox et al., 2024), which is consistent with previous results (Liao et al., 2015). The treatment group also displayed greater white matter and corpus collosum preservation compared to controls after 1 year post-treatment (Cox et al., 2024).

A number of clinical trials are currently ongoing. A phase 2 clinical trial (NCT05951777) scheduled for completion in 2026 will investigate the safety of autologous AD-MSCs in patients with TBI. Another phase 1 trial (NCT05018832) scheduled to be completed by 2028 will investigate the safety of allogenic hUC-MSCs in patients with chronic TBI. Finally, a phase 1/2 trial investigating autologous AD-MSCs (NCT04063215) was recently completed in 2024, but results are still under review.

It must be noted that acute and chronic phase TBI are distinguished by unique mechanisms of cell death and neuroinflammation and may respond to MSC therapy differentially. The majority of clinical trials have focused on chronic phase TBI, whereas fewer have focused on acute TBI, and only one completed study has focused on acute TBI in adults, though we are still awaiting results from NCT02525432. Similarly to stroke, it is logistically challenging to intervene in the acute phase of injury, but evidence from preclinical studies shows that expeditious administration of cell-based therapies results in greater clinical recovery. Table 7 describes completed and ongoing clinical trials of MSCs in TBI.

**TABLE 7:** Clinical trials investigating MSC-based therapies for TBI.

Trial ID, status, phase	Study design	# PPTS	Cell-type and route of administration	Intervention	Results	Citation
NCT00254722, Completed, Phase 1	Open label, single arm	10 patients 5–14 years old with TBI < 48 h and GCS between 5 and 8	Autologous bone marrow mononuclear cells (BMMNCs), Intravenous	1 infusion of 6.0 * 10^6^ cells/kg	No treatment related toxicity. Dichotimmized Glasgow Outcome Score at 6 months showed 70% with good outcomes and 30% with moderate to severe disability.	(Cox et al., 2011)
NA, Completed, -	Randomized, single blind, controlled, parallel assignment	40 patients w/Sequelae of TBI (> 1 year)	Allogenic hUC-MSCs, Intrathecal	4 injections 1.0 * 10^7^ cells, once every 5–7 days	Improvements in FMA and FIM scores in MSC treatment group compared to controls	(Wang et al., 2013)
NA, Completed, Phase 1	Open label, single arm	14 patients w/chronic TBI	Autologous BMMNCs, Intrathecal	1.0 * 10^6^ cells/kg + neurorehabilitation.	Majority of patients experienced improvements in balance, voluntary control, memory, oromotor activity, upper limb and trunk movement, and ambulation.	(Sharma et al., 2015)
NCT01575470, Completed, Phase 1/2	Non-randomized, placebo-controlled, dose escalation study	25 adult patients w/Acute TBI (< 24 h prior to consent) and GCS between 5 and 8	Autologous BMMNCs, Intravenous	1 infusion. Low dose group: 6.0 * 10^6^ cells/kg. Mid dose group: 9.0 * 10^6^ cells/kg. High dose group: 12.0 * 10^6^ cells/kg	No serious adverse events observed. Treated groups showed lower levels of TNF-a, IL-1β, IL-4, IL-6, and IFN-y.	(Cox et al., 2017)
NCT02416492, Completed, Phase 2	Randomized, double blind, parallel assignment, placebo controlled	63 patients w/TBI > 12 months	SB623 ^®^ (Allogenic BM-MSCs transfected with Notch-1), Stereotactic Implantation	2.5, 5.0, or 10.0 * 10^6^ cells	Significant improvement in FMMS at 6 months compared to controls	(Kawabori et al., 2021)
NCT01851083, Completed, Phase 2	Randomized, double blinded, placebo controlled parallel assignment, dose escalation	47 patients age 5–17 w/acute TBI (< 48 h) and GCS 3–8	Autologous BMMNCs, Intravenous	1 infusion of either 6.0 * 10^6^ cells/kg or 10 * 10^6^ cells/kg	Improved structural preservation in the brain. Shorter and less medically intensive stays in the ICU	(Cox et al., 2024)
NCT04063215, Completed, Phase 1/2	Open label, single arm	24 patients w/head injury and documented damage to the CNS. GOS-E between 2 and 6	Autologous AD-MSCs, Route Unspecified	3 infusions of 2.0 * 10^8^ cells	Not yet published	N/A
NCT02525432, Active, Not Recruiting, Phase 2	Randomized, double blind, placebo controlled, parallel assignment, dose escalation	37 patients w/Acute TBI (< 48 h). GCS between 3 and 8	Autologous BMMNCs, Intravenous	1 Infusion. group 1: 6.0 * 10^6^ cells/kg. Group 2: 9.0 * 10^6^ cells/kg	–	N/A
NCT05951777, Recruiting, Phase 2	Randomized, double blind, placebo controlled, parallel assignment	51 patients w/TBI. GOS-E between 2 and 6	Autologous AD-MSCs, Intravenous	3 infusions of 2.0 * 10^8^ cells at 14 day intervals.	–	N/A
NCT05018832, Not Yet Recruiting, Phase 1	Open label, single arm	20 patients w/TBI	Allogenic, hUC-MSCs, Intravenous	1 infusion of 1.0 * 10^8^ cells	–	N/A

Data in this table were gathered using clinicaltrials.gov (Clinicaltrials.gov, 2025) or through consulting the corresponding reference listed in the table. References are provided only if the clinical trial results have been published. Note: clinicaltrials.gov is not always up to date. Presented data reflect corresponding publications where available. GCS, Glasgow Coma Scale; FMA, Fugl-Meyer Assessment; FIM, Functional Independence Measure; FMMS, Fugl-Meyer Motor Scale; GOS-E score, Glasgow Outcome Scale-extended score. Efforts were made to include completed trials as well as those in the “active, not recruiting,” “recruiting” and “not yet recruiting” phases. Clinical trials with unknown status are included when appropriate.

### 3.8 Degenerative disc disease

Disease and injury of the spine itself and intervertebral discs can greatly affect the CNS, causing disability, pain, loss of sensation, and loss of function. MSCs have shown potential in treating degenerative disc disease (DDD), a common cause of lower back pain (LBP) which can damage the nerves of the spinal cord (Richardson et al., 2016; Lim et al., 2017).

The intervertebral disc is composed of type II collagen, proteoglycans, and type I collagen, conferring flexibility and strength crucial for spinal support and everyday motion (Richardson et al., 2016). On a pathological level, intervertebral disc degeneration is characterized by an increase in inflammatory cytokines TNF-α, IFN-γ, IL-6, and IL-1β, released by the nucleus pulposus (NP), the central structure of the disc (Shamji et al., 2010; Richardson et al., 2016). Histological evidence also suggests a role for macrophages, Th1 and Th17 lymphocytes in the pathology of DDD (Shamji et al., 2010). Additionally, increased levels of IL-1β in DDD are associated with reduction in NP water content and cell count, in part mediated by MMPS (Le Maitre et al., 2005; Le Maitre et al., 2007; Hoyland et al., 2008; Liang et al., 2022). Increased MMP activity is associated with alternative expression of collagens I and III in the NP and reduced synthesis of aggrecan (Le Maitre et al., 2007), thus leading to dehydration and increased mechanical stress on the disc (Liang et al., 2022).

#### 3.8.1 MSC preclinical trials in DDD

BM-MSCs and AD-MSCs are capable of differentiating into the NP phenotype (Richardson et al., 2006; Clarke et al., 2014; Richardson et al., 2016). A number of preclinical trials have shown that MSC transplantation in DDD can increase aggrecan expression and matrix production (Sakai et al., 2005; Feng et al., 2011; Chun et al., 2012; Marfia et al., 2014), which may lead to an increase in disc height due to rehydration of the disc (Jeong et al., 2009). MSC transplantation may also induce NP cells to suppress MMP and inflammatory cytokine genes (Miyamoto et al., 2010). Altogether, these preclinical trials suggest that MSCs target the deleterious mediators of inflammation and matrix destruction as well as promote matrix repair and rehydration.

MSC-derived exosomes alone also exert many of the same effects, increasing cell proliferation, hydration and extracellular matrix deposition while reducing oxidative stress, attenuating inflammation, and preventing apoptosis of cells within the disc (Bhujel et al., 2022). Additionally, pre-treatment of hUC-MSCs to induce a chondrogenic progenitor-like phenotype showed enhanced improvement in DDD compared to untreated MSCs, potentially due to increased ability to differentiate into NP cells and ability to survive in the relatively hypoxic environment of the intervertebral disc (Ekram et al., 2021).

#### 3.8.2 MSC clinical trials in DDD

Clinical trials in DDD have demonstrated that BM-MSCs, and AD-MSCs are safe and quite effective in treating DDD. The first in-human evidence for the efficacy of MSCs in treating LBP was provided by Yoshikawa et al. (2010). 2 patients with lumbar spinal canal stenosis underwent surgical implantation of collagen sponges loaded with autologous BM-MSCs into the affected lumbar discs. The patients experienced lasting pain relief at 2 years post-surgery and notable disc rehydration on T2-weighted MRI (Yoshikawa et al., 2010).

Orozco et al. followed this up with a larger study in 10 patients with LBP due to lumbar disc degeneration (Orozco et al., 2011). Patients were treated with autologous BM-MSCs and reduction in pain and disability was measured via the visual analog scale (VAS) and the Oswerty Disability Index (ODI) respectively. Participants experienced reduction in LBP as early as 3 months post-treatment which persisted through 1 year of follow-up. They also experienced decreased disability status as assessed by the ODI. Though there was no improvement in disc height, the authors observed an increase in fluid content of the treated discs (Orozco et al., 2011). The strong safety and efficacy results of this clinical trial proved the feasibility of MSC transplantation for DDD and suggested that MSCs rehydrate degenerating discs, which is consistent with the data from Yoshikawa et al. (2010) and previous preclinical trials (Jeong et al., 2009; Marfia et al., 2014)

The same research group conducted a follow-up phase 1/2 clinical trial (NCT01860417) from 2013-2015 to test the efficacy of allogenic BM-MSCs, rather than autologous BM-MSCs, in the treatment of DDD (Noriega et al., 2017). Pain scores on the VAS improved at 3 months but declined from peak relief by 12 months. ODI scores, while improved, did not match improvements observed from autologous BM-MSCs (Orozco et al., 2011; Noriega et al., 2017). However, a subsequent long-term follow-up study from this trial reported that pain and disability scores continued to improve as long as 3.5 years after treatment in those patients administered allogenic BM-MSCs compared to controls (Noriega et al., 2021), altogether providing convincing evidence for the long-term benefits of MSCs used to treat DDD.

A phase 1/2 clinical trial (NCT01513694) recently demonstrated the benefit of autologous BM-MSCs embedded within tricalcium phosphate for spinal fusion surgery outcomes. Patients underwent MSC implantation during posterolateral spinal fusion surgery and were monitored for 12 months. MSCs were shown to aid in L4-L5, and L5-S1 spinal fusion with no adverse events. VAS and ODI scores also improved after the surgery, and 9 patients demonstrated successful solid fusion of the spine (Blanco et al., 2019).

MSCs have also been studied in combination with plate-rich plasma (PRP), an autologous mixture of platelets, trophic factors, and cytokines already used as a treatment for DDD and other joint pathologies (Kawabata et al., 2023). A pilot study by Comella et al. (NCT02097862) recruited 15 patients with lumbar DDD and treated them with autologous AD-MSCs and PRP. The treatment was found to be safe and modestly effective in improving flexion and reducing subjective measures of pain (Comella et al., 2017). However, more studies are needed to characterize the cooperative effect of MSCs with PRP.

The strongest evidence supporting the therapeutic benefit of MSCs in DDD comes from a recently completed phase 3 trial (NCT02412735) conducted in 404 patients with chronic LBP. Patients were administered allogenic bone marrow-derived MSCs with or without hyaluronic acid. While the primary and secondary efficacy endpoints were not met, the investigators reported a strong reduction in LBP and ODI disability scores in all treatment groups. Furthermore, MSC + hyaluronic acid treatment resulted in a substantial reduction in LBP, especially in patients with LBP less than 68 months. In these patients, MSC treatment was also associated with greater functional improvement, quality of life, and less opioid usage. Though primary efficacy endpoints were not met, the results of this trial are promising and warrant further exploration in patients with chronic LBP less than 68 months (Beall et al., 2025).

A number of upcoming clinical trials for MSCs in DDD have been initiated. A phase 2 study (NCT004042844) in 99 patients with suspected discogenic back pain will administer autologous BM-MSCs cultured in hypoxic media, thus acclimating them to the physiologic environment of the intervertebral disc. Another phase 2 study (NCT05066334) will enroll 52 patients with DDD at multiple lumbar discs. Autologous MSCs will be administered to up to 3 disc segments.

Overall, these studies provide strong evidence that MSC can reduce pain, improve disability, and elicit disc rehydration in DDD. Table 8 includes a brief summary of clinical trials in DDD.

**TABLE 8:** Clinical trials investigating MSC-based therapies for DDD.

Trial ID, status, phase	Study design	# PPTS	Cell-type, route of administration	Intervention	Results	Citation
NA, Completed, –	Open label, single arm	2 patients w/lumbar spinal canal stenosis	Autologous BM-MSCs, Intradiscal	Collagen sponge loaded with 1.0 * 10^5^ cells	Both patients showed improvement in symptoms and improved hydration of disc on CT and MRI imaging after 2 years	(Yoshikawa et al., 2010)
NA, Completed, Phase 1	Open label, single arm	10 patients w/chronic LBP due to lumbar disc degeneration	Autologous BM-MSCs, Intradiscal	1 Injection of 10 ± 5 * 10^6^ cells/disc	Treatment was safe and patients experienced rapid improvement in pain and disability. No change in disc height, but increased fluid content was observed.	(Orozco et al., 2011)
NCT02097862, Completed, Phase NA	Open label, single arm	15 patients w/lumbar DDD at 1, 2, or 3 disc segments	Stromal Vascular fraction of Adipose tissue (Autologous AD-MSCs), Intradiscal	1 injection of between 30 and 60 * 10^6^ cells in autologous platelet rich plasma (PRP) medium.	No severe Adverse events reported. Improvements in flexion range and pain on VAS	(Comella et al., 2017)
NCT01860417, Completed, Phase 1/2	Randomized, double blind, placebo controlled, parallel assignment	24 patients w/LBP from lumbar disc degeneration	Allogenic BM-MSCs, Intradiscal	1 injection of 25 * 10^6^ cells/disc	No major adverse events. Subset of treated group experienced pain reduction at 3 months that did not improve further. Statistically significant improvement in ODI.	(Noriega et al., 2017)
NCT01513694, Completed, Phase 1/2	Open label, single arm	11 patients w/DDD at L4-L5 or L5-S1	Autologous BM-MSCs, Implantation during posterolateral spine fusion surgery	Mixture of 0.5–1.5 * 10^6^ cells/kg and tricalcium phopshate	No treatment-related adverse events. Both VAS and ODI improved in treated patients. Solid fusion observed in 9 patients	(Blanco et al., 2019)
NCT02412735, Completed, Phase 3	Randomized, double blind, placebo-controlled, parallel assignment	404 patients w/LBP due to DDD between L1-S1	Rexlemestrocel-L ^®^ (Allogenic BM-MSC), Intradiscal	Group 1: ∼6.0 * 10^6^ cells. Group 2: ∼6.0 * 10^6^ cells + Hyaluronic acid	Overall primary and secondary efficacy endpoints not met. However, treatment groups improved from baseline	(Beall et al., 2025)
NCT04042844, Recruiting, Phase 2	Randomized, double blind, parallel assignment, placebo controlled	99 patients w/chronic LBP due to DDD	BRTX-100 ^®^ (Hypoxic cultured autologous BM-MSCs), Intradiscal	1 injection of 40.0 * 10^6^ cells.	-	N/A
NCT05066334, Recruiting, Phase 2	Randomized, Double blind, placebo-controlled, parallel assignment	52 patients w/chronic LBP due to multi-level DDD	Autolgous BM-MSCs, Intradiscal	15.0 * 10^6^ cells/disc, up to 3 discs.	-	N/A

Data in this table were gathered using clinicaltrials.gov (Clinicaltrials.gov, 2025) or through consulting the corresponding reference listed in the table. References are provided only if the clinical trial results have been published. Note: clinicaltrials.gov is not always up to date. Presented data reflect corresponding publications where available. DDD, Degenerative disc disease; LBP, Lower back pain; VAS, Visual analog scale of pain; ODI, Oswerty Disability Index. Efforts were made to include completed trials as well as those in the “active, not recruiting,” “recruiting,” and “not yet recruiting” phases. Clinical trials with unknown status are included when appropriate.

### 3.9 Sepsis/meningitis

The conditions discussed so far in this review are largely the result of neurodegeneration, autoimmunity, or injury, but infection, specifically sepsis and meningitis, represents a significant threat to neurologic health. Sepsis refers to organ dysfunction caused by widespread immune system dysregulation in response to an infection. Traditional definitions of sepsis characterized it as an inappropriately exaggerated inflammatory response to infection, but contemporary evidence has suggested that it may be better understood as an interplay of pro- and anti-inflammatory states which precipitate widespread organ dysfunction and death (Remick, 2007; Shankar-Hari et al., 2016; Laroye et al., 2017). It is estimated to affect around 50 million people globally each year and is a major contributor to mortality (Rudd et al., 2020).

Sepsis is a frequent causative factor meningitis, especially in infants (Overturf, 2003; Kim F. et al., 2020), because a major route of bacterial CNS infiltration is through the BBB during episodes of bacteremia. However, meningitis can also occur in the absence of sepsis through contiguous spread (van de Beek et al., 2016). Meningitis refers to the specific inflammation of the meninges, a collection of 3 tissue layers which surround the brain consisting of the dura mater, the arachnoid mater, and the pia mater (Poplin et al., 2020). This inflammation can even extend to the brain parenchyma itself (van de Beek et al., 2016). The most common causes of bacterial meningitis include *Streptococcus pneumoniae, Haemophilus. Influenzae, Neisseria meningitidis*, and *Escherichia coli* (Quagliarello and Scheld, 1992).

The pathogenesis of sepsis and meningitis are quite similar with the primary difference being that meningitis takes place within the CNS (Leib and Täuber, 1999). In both conditions the host immune system recognizes an invading pathogen and triggers a response, which is largely comprised of lymphocyte recruitment, activation of the complement system, and release of inflammatory mediators like TNF, IL-1β, IL-12, IL-18, and ROS, among others. Tissue damage that occurs due to infection or inflammation releases damage-associated molecular patterns (DAMPs) which further exacerbate the inflammatory response (Leib and Täuber, 1999). Concurrently, immune suppression occurs due to elevated anti-inflammatory cytokine release, reduced inflammatory cytokine production, and cellular immune exhaustion (van der Poll et al., 2021).

#### 3.9.1 MSC preclinical trials in sepsis/meningitis

MSCs have already been shown to provide benefit in preclinical studies of sepsis (Krasnodembskaya et al., 2010; Mei et al., 2010; Jackson et al., 2016; Laroye et al., 2017; Morrison et al., 2017). The therapeutic benefit of MSCs in sepsis is thought to be derived from modulation of immune cells, promotion of bacterial autophagy and reduction of inflammatory mediators (Krasnodembskaya et al., 2010; Mei et al., 2010; Laroye et al., 2017). Both BM-MSCs and hUC-MSCs were found to induce CD3 + CD4 + and CD25 + T regulatory cells, which coincided with a downregulation in IL-6 and TNF-α (Chao et al., 2014)., suggesting potent immune regulation and suppression of damaging immune responses.

Another potential mechanism is the transfer of mitochondria from MSCs to host macrophages, thereby aiding the process of autophagy (Jackson et al., 2016; Morrison et al., 2017). These same mechanisms also enable MSCs to decrease the risk of organ failure from sepsis and septic shock (Laroye et al., 2017).

Preclinical models of meningitis have provided evidence for the beneficial effect of MSCs, and most have come from models of neonatal meningitis. Transplantation of human umbilical cord blood-derived MSCs in a newborn rat model of meningitis resulted in reduced bacterial growth rate and mortality. Levels of inflammatory cytokines IL-1α, IL-1β, IL-6, and TNF-α as well numbers of activated macrophages were reduced. Additionally, functional behavioral tests improved at a faster rate in rats treated with MSCs (Ahn et al., 2018). Additionally, concomitant treatment of bacterial meningitis in newborn rats with MSC-exosomes did not enhance bacterial clearance but did reduce neuroinflammation and neural apoptosis (Kim et al., 2022), suggesting that MSC exosomes exert a neuroprotective effect, but are not as effect in clearing the infection as MSCs themselves.

The source of MSCs appears to significantly influence outcomes in animal models of sepsis. A study directly comparing human BM-MSCs against hUC-MSCs showed significant improvement in survival and bacterial clearance in the BM-MSC-treated mice, but not in the hUC-MSC-treated mice. 7-day survival rate for mice treated with BM-MSCs was 64.71%, while the survival rate in the hUC-MSC group was 29.4% (Varkouhi et al., 2021). However, there is conflicting evidence that BM-MSCs and UC-MSCs are equally effective in regulating the immune response in animal models of sepsis (Chao et al., 2014), especially when administered within the right time frame (Gonzalez et al., 2020).

Models of sepsis also show improvement when treated with MSC-derived exosomes. Exosomes of MSCs have been shown to reduce bacterial load in sepsis-induced lung injury, kidney injury, cardiovascular injury, and liver injury (Cheng Y. et al., 2020). The improvement was found to be due in part to delivery of mRNA and miRNA to injured tissue, thereby increasing gene expression and enhancing phagocytosis of bacteria by macrophages and promoting an anti-inflammatory M1 phenotype while reducing cellular apoptosis (Lee et al., 2013; Monsel et al., 2015; Zhu et al., 2019). These studies suggest that MSC-derived exosomes may be more effective than whole-cell therapy in the treatment of sepsis due to their ability to deliver factors directly to target cells. Treatment of sepsis requires timely administration of therapy, and exosomes are stable when stored, allowing for quick use and lack of immunogenicity where allogenic MSCs may exacerbate an already unstable immune system (Cheng Y. et al., 2020).

#### 3.9.2 MSC clinical trials in sepsis/meningitis

Several clinical trials have proven the safety of MSCs in treating sepsis (Khosrojerdi et al., 2021). Two early phase 1 clinical trials conducted in the 2010s using allogenic MSCs reported good safety profiles in patients under septic shock (Galstian et al., 2015; McIntyre et al., 2018) with another phase 1/2 study having demonstrated improved short-term survival in patients treated up to 5 times with allogenic AD-MSCs within 9 days (Alp et al., 2022). Looking forward, one phase 2 study (NCT05969275) hopes to recruit 296 patients and investigate whether allogenic hUC-MSC transplantation reduces the need for ventilator support, renal replacement therapy, and vasopressors. Another phase 1 study (NCT06882811) aims to determine whether allogenic hUC-MSCs improve mortality rate at 28 days post-treatments.

While the therapeutic use of MSCs for sepsis has been widely characterized, it has been less well-studied in meningitis. Preclinical studies have demonstrated therapeutic potential (Ahn et al., 2018; Kim et al., 2022), but there have been no clinical trials for meningitis and MSCs registered on clinicaltrials.gov. However, given the pathological similarities between sepsis and meningitis, it is reasonable to think that MSCs could be safely and effectively used to ameliorate harmful inflammatory responses in meningitis as well as sepsis. Due to the heterogenous nature of sepsis and meningitis, a unified treatment has not yet been developed, but MSCs present a novel therapeutic opportunity for treating both sepsis and meningitis. Table 9 presents data from clinical trials in patients with sepsis treated using MSCs.

**TABLE 9:** Clinical trials investigating MSC-based therapies for sepsis and meningitis.

Trial ID, status, phase	Study design	# PPTS	Cell-type and route of administration	Intervention	Results	Citation
NCT01849237, Completed, Phase 1/2	Randomized, open-label, parallel assignment	30 patients w/septic shock and severe neutropenia	Allogenic BM-MSCs, Intravenous	1 infusion of 1.0 * 10^6^ cells/kg within first 10 h of septic shock onset	Patients treated with MSCs experienced significantly greater rates of short term survival (28 days) but not long term survival	(Galstian et al., 2015)
NCT02421484, Completed, Phase 1	Open label, single arm, dose escalation study	9 patients w/septic shock	Allogenic BM-MSCs, Intravenous	Low dose: 0.3 * 10^6^ cells/kg. Medium dose: 1.0 * 10^6^ cells/kg. High dose: 3.0 * 106 cells/kg	Allogenic BM-MSCs is safe and feasible for septic shock	(McIntyre et al., 2018)
NCT05283317, Completed, Phase 1/2	Non-randomized, single blind, parallel assignment	30 patients w/sepsis and septic shock	Allogenic AD-MSCs, Intravenous	5 infusions of 1.0 * 10^6^ cells/kg at 2 day intervals.	Treatment was well tolerated with no treatment-related adverse effects. MSC treatment improved short term survival (28 days)	(Alp et al., 2022)
NCT04080921, Completed, Phase 1/2	Open label, single arm	22 patients w/neurological sequalae from meningitis or Encephalitis	Autologous BMMNCs, Intrathecal	2 injections at baseline and at 6 months. Dosage unspecified	Not yet published	N/A
NCT05969275, Recruiting, Phase 2	Randomized, double blind, placebo-controlled, parallel assignment	296 patients w/septic shock.	Allogenic hUC-MSCs, Intravenous	1 infusion of 300 * 10^6^ cells.	–	NA
NCT06882811, Recruiting, Phase 1	Randomized, double blind, parallel assignment, placebo controlled	180 patients w/sepsis	Allogenic hUC-MSCs vs. CD38 + MSCs, Intravenous	Group 1: 1.0 * 10^8^ of CD83 + MSCs. Group 2: 1.0 * 10^8^ hUC-MSCs	–	NA

Data in this table were gathered using clinicaltrials.gov (Clinicaltrials.gov, 2025) or through consulting the corresponding reference listed in the table. References are provided only if the clinical trial results have been published. Note: clinicaltrials.gov is not always up to date. Presented data reflect corresponding publications where available. Efforts were made to include completed trials as well as those in the “active, not recruiting,” “recruiting,” and “not yet recruiting” phases. Clinical trials with unknown status are included when appropriate.

## 4 MSC allograft vs. autograft

Autografts refer to tissue samples administered to the same host from which they were obtained (Miller et al., 2011), whereas allografts refer to tissue samples taken from one organism and transplanted into another organism of the same species (Ceuppens and Goffin, 2017). In many conditions that require grafting, autografts are favored for their negligible immunogenicity, but drawbacks include availability of host tissue and risks of obtaining the autograft tissue (Malloy and Hilibrand, 2002; Deutsch et al., 2007; Baldwin et al., 2019). Allografts have historically posed a greater risk of provoking a host immune response, leading to allograft rejection in some cases (Aubert et al., 2024), but MSC allografts have been shown to be significantly less immunogenic than other allografts (Barry et al., 2005). Allogenic MSCs were considered “immune-privileged” for a time, but current evidence suggests that allogenic MSCs do indeed provoke an immune response *in vivo* (Berglund et al., 2017). Despite their potential downsides, allogenic MSC treatments carry a unique advantage in their “off-the-shelf” availability, whereas autologous MSC treatments require time to procure and expand (Li et al., 2021b).

There is ongoing debate as to whether allogenic MSC transplants demonstrate similar safety and efficacy profiles to autologous MSC treatments (Hare et al., 2012; Hare et al., 2017; Li et al., 2021b). Animal studies comparing autologous and allogenic MSC transplantations have been conducted. One study in a canine model of SCI showed that both allogenic and autologous MSCs improved functional outcomes, but numbers of allogenic MSCs were diminished at 4 weeks post-injury compared to the autologous MSCs (Jung et al., 2009), suggesting that allogenic MSCs may not survive for as long as autologous MSCs *in vivo.*

There is also the matter of MSC immunogenicity. MSCs express MHC I, and under certain conditions can express MHC II surface proteins (Galipeau and Sensébé, 2018). While MSCs tend to provoke a host immune response far less than other treatments they are not entirely immune-privileged (Ankrum et al., 2014; Berglund et al., 2017), and evidence from Reinders et al. suggests that allogenic MSCs elicit an immune response in kidney transplant patients (Reinders et al., 2015). However, the alternative of autologous MSC transplant may be unfeasible for certain conditions that require acute treatment, like stroke, and allogenic MSC treatment may still provide a benefit (Galipeau and Sensébé, 2018).

Another aspect that must be considered in the autograft/allograft debate is autoimmune conditions. In individuals with autoimmune conditions, like MS, autografts may not be as effective as allografts due to altered senescence, gene expression, and morphology (de Oliveira et al., 2015; Redondo et al., 2018; Sarkar et al., 2018). The same is true of patients with systemic lupus erythematosus and systemic sclerosis (Rozier et al., 2018; Cheng et al., 2019). Thus, despite their immunogenicity, allogenic MSCs may be the optimal choice for patients with autoimmune conditions.

## 5 Future approaches for MSCs

Given the inconsistency of clinical trial results, MSC therapies have a long way to go in terms of clinical efficacy. As a result, several avenues have been taken to enhance MSC therapeutics. For instance, culturing MSCs under quasi–*in vivo* conditions could promote their survival and prolong their therapeutic effect (Liu et al., 2013; Levy et al., 2015; Pittenger et al., 2019). MSCs cultured in a low oxygen environment (5% oxygen) showed increased potency and fitness when compared to MSCs cultured in atmospheric conditions (20% oxygen) (Haque et al., 2015; Elabd et al., 2018; Hu and Li, 2018). The same is true of MSCs preconditioned with various pharmaceutical agents and inflammatory cytokines (Carrero et al., 2012; François et al., 2012; Hu and Li, 2018). One such example of preconditioned MSCs are the NurOwn^®^ cells which have been used in several clinical trials (Berry et al., 2019; Cudkowicz et al., 2022; Cohen et al., 2023). These cells are cultured in a specific medium-based approach to overproduce BDNF, GDNF, VEGF, and HGF (Gothelf et al., 2017), enabling them to secrete trophic factors that both enhance host mechanisms of repair and promote their own survival. NurOwn^®^ cells have a distinct miRNA profile compared to other MSCs, with upregulated miRNAs linked to repression of proliferative and anti-apoptotic genes, and downregulated miRNAs associated with neurogenesis and CNS development (Gothelf et al., 2017).

Genetic modification of MSCs is another promising avenue for future research but has so far been studied nearly exclusively in animal models (Pawitan et al., 2020). MSCs can be genetically modified to overexpress GDNF, NGF, and BDNF, which have broad applicability in a number of neurodegenerative disorders (Wyse et al., 2014). They can also be tailored for more specific uses. For example, MSCs genetically modified to express genes related to dopamine synthesis were found to ameliorate Parkinson’s symptoms in rats (Li J. et al., 2022). Inducing MSCs to overexpress IL-10 in a rat model of TBI significantly improved recovery and reduced histological markers of inflammation (Peruzzaro et al., 2019). Genetically engineering MSCs to express chABC, an enzyme which cleaves the CSPG scar around spinal cord lesions (Silver and Miller, 2004), can improve recovery from SCI (Prager et al., 2021). Additionally, MSC homing to sites of injury can be enhanced through expression of FGF21 and CXCR4 genes (Shahror et al., 2020). A few clinical trials using genetically edited MSCs have been initiated in lung cancer, (TACTICAL trial NCT03298763), and gastrointestinal cancer (TREAT-ME trial, NCT02008539) patients, though the former is in the recruiting phase, and the latter was terminated due to an inclusion default. To our knowledge, no human clinical trials of neurological disease or injury involving genetically edited MSCs have been initiated, but results from preclinical studies indicate such therapies are promising (Wyse et al., 2014; Prager et al., 2021; Li J. et al., 2022).

Culturing MSCs on bio-scaffolds has been shown to enhance cell survival and reduce oxidative stress (Liu et al., 2016). Similarly, 3D culturing techniques, such as micro-well, hanging drop, and ultra-low attachment plate-based spheroid methods, more closely replicate the *in vivo* environment, resulting in increased MSC pluripotency and greater secretion of trophic and anti-inflammatory cytokines (Foty, 2011; Kim et al., 2019; Kouroupis and Correa, 2021; Thakur et al., 2022). Clinical trials using bio-scaffolds have reported some success, especially in SCI (Zhao et al., 2017; Chen W. et al., 2020), but larger phase 3 studies are needed to fully characterize their effects in humans.

The loading of MSCs with nanoparticles of various types has shown promise in potentiating MSC therapies. Kim et al. showed that pre-treatment of autologous BM-MSCs with iron oxide nanoparticles (IONP) produced magnetic exosomes which could be guided to target tissues via magnetic field. The exosomes treated with IONPs demonstrated increased levels of growth factors, including BDNF, VEGF, and NGF. A mouse MCAO stroke model treated with these magnetic exosomes showed significantly reduced infarct volume and functional improvement (Silver and Miller, 2004). Furthermore, this approach is biocompatible because excess iron oxide can be metabolized and stored as ferritin (Kim H. Y. et al., 2020). Another study took a similar approach but administered whole human WJ-MSCs loaded with IONPs to a mouse model of AD through intracerebroventricular injection. Application of external magnetic field retained MSCs in the brain. Additionally, WJ-MSCs treated with IONPs produced high levels of anti-inflammatory cytokines and growth factors, such as BDNF, NGF, NT-3, and TGF-β1, mediated through the c-Jun N-terminal kinase (c-JNK) signaling pathway (Jung et al., 2023). Similarly, human BM-MSCs treated with dextran-coated IONPs improved functional recovery in a mouse model of PD (Chung et al., 2018). Nanoparticles have also been used to protect MSCs from the hostile inflammatory environment of the injured CNS. Wu et al. demonstrated that NaGdF_4_ nanoparticles coated with polydopamine and alendronate protected BM-MSCs from ROS and calcium overload in the post-stroke inflammatory milieu of the brain in a MCAO mouse model of stroke (Wu S. et al., 2025). Despite their potential, nanoparticles may be cytotoxicity at higher concentrations, which necessitates further investigation of optimal nano-particle dosing before translational trials (Kim H. Y. et al., 2020; Jung et al., 2023; Wu S. et al., 2025).

Yet another emerging technology which could optimize the potency of MSC therapies is the application of electromagnetic fields (EMF). Low frequency EMFs (between 0 and 100 Hz) can affect a wide range of biological functions, such as gene expression, cell differentiation, protein metabolism, and even stem cell differentiation (Safavi et al., 2022). The human body naturally produces EMFs as a result of bioelectric processes which move ions across cell membranes and influence membrane potentials (Levin, 2009). Exogenous EMFs can modulate bioelectric signaling and thus alter gene expression and cellular pathways which can influence stem cell differentiation (Tamrin et al., 2016; Safavi et al., 2022). Pulsed EMF waves were shown to increase chondrogenesis in MSCs through release of secreted factors possibly through interacting with cellular calcium permeability (Parate et al., 2017; Parate et al., 2020) and influencing mitochondrial metabolism (Yap et al., 2019). Several other studies have demonstrated that pulsed EMFs improved the osteogenic differentiation potential of both BM-MSCs and AD-MSCs (Wang H. et al., 2019; Aldebs et al., 2020). Most importantly for conditions of the CNS, EMFs have been shown to positively influence neurogenic and astrocyte differentiation of MSCs even in the absence of growth factors or pharmacologic factors (Cho et al., 2012; Aikins et al., 2017; Jeong et al., 2017; Seo et al., 2018). To our knowledge, the use of EMFs to potentiate MSCs remains unexplored in humans but represents a promising technology to enhance MSC therapies (Safavi et al., 2022).

Ultrasound can also be used to enhance the therapeutic efficacy of MSCs. Ultrasound exerts a mechanical pressure on cells when used at frequencies between 0.02 and 3.0 MHz, inducing mechanical transduction cascades which can influence gene expression (Liu et al., 2020). Interestingly, ultrasound can be applied directly to MSCs to enhance differentiation as well as to target tissue to increase MSC tropism through upregulation of homing factors like cytokines, adhesion molecules, and growth factors (Liu et al., 2020). Low frequency ultrasound combined with human BM-MSCs in a rat model of stroke reduced infarct size and increased neural protein expression compared to controls (Cho et al., 2016). Focused ultrasound has also been used to transiently disrupting the BBB and enhance extravasation of MSCs to site of injury within the brain parenchyma (Joshi et al., 2011; Wang et al., 2022; Wu S. K. et al., 2025). In a rat MCAO model of stroke, this approach led to significant functional improvements and enhanced migration of BM-MSCs to the infarcted tissue compared to BM-MSCs without ultrasound (Qian et al., 2019). Furthermore, extracorporeal shock wave therapy, a type of high intensity ultrasound technique originally used to disintegrate kidney stones (Mittermayr et al., 2012), has been shown to improve engraftment of autologous BM-MSCs in the spinal cord in a rat model of SCI (Lee et al., 2014).

Ultimately ultrasound techniques and nano-particle-loaded MSCs aim to improve cell delivery to target tissues, while genetic editing of MSCs, pretreatment with EMFs, use of bio-scaffolds, and culturing in inflammatory environments helps enhance cell survival once there. A combined approach may yield the greatest efficacy, but more research is needed to optimize individual techniques and fully characterize their effects before a combined approach is investigated.

## 6 The neural exposome and MSC therapy

The exposome, a concept introduced by Christopher Wild in his 2006 publication, refers to the totality of environmental exposures across an individual’s lifespan which act alongside the genome to influence risk and morbidity of disease (Wild, 2005). The neural exposome refers to non-genetic factors that exert an effect on the CNS, such as the microbiome, environmental toxins, climate, infections, socioeconomic status and education, psychological health and stress, drug use, lifestyle, and sleep, among others (Tamiz et al., 2022; Sakowski et al., 2024). The neural exposome interacts with an individual’s genome to influence risk for certain neurological conditions (Migliore and Coppedè, 2022; Sakowski et al., 2024), especially neurodegenerative conditions like PD, ALS, and AD (Sakowski et al., 2024). For example, individuals with the rs1803274 single nucleotide polymorphism in the *BCHE* gene encoding for the bioscavenger butyrylcholinesterase who are exposed to pesticides are at increased risk of developing PD (Tufail, 2019). The mechanisms of action by which environmental exposures such as air pollution, pesticide exposure, and metals exert their effects include neuroinflammation, microglial activation, mitochondrial and oxidative stress, and abnormal protein accumulation, all of which are known pathologic mechanisms of neurodegeneration (Calderón-Garciduenas et al., 2008; Parrón et al., 2011; Chin-Chan et al., 2015; Sakowski et al., 2024).

While the neural exposome has yet to be investigated in the context of stem cell therapies, there is evidence that heavy exposure to environment factors which affect brain health lead to worse outcomes in neurologic and neurodegenerative diseases (Chin-Chan et al., 2015; Gunnarsson and Bodin, 2019; Giovannoni et al., 2024). Perhaps unsurprisingly, evidence also suggests that fostering an enriching environment and encouraging positive lifestyle modifications positively influences recovery and reduces progression in SCI, stroke and MS (McDonald et al., 2018; De Virgiliis et al., 2020; Han et al., 2023; Simpson-Yap et al., 2023; Hedström et al., 2025). Such evidence suggests that combining MSC therapies with lifestyle modifications may improve outcomes.

No direct evidence exists confirming that environmental exposures reduce responsiveness to MSC therapies, owing in part to the fact that very few MSC therapies are approved worldwide. Their interaction with environmental risk factors is insufficiently explored, but one thing is clear: MSCs are environmentally responsive entities, meaning that they will behave differently depending on their immediate biological environment (Murphy et al., 2013). For example, inflammation in the body, triggers the secretion of anti-inflammatory factors from MSCs, including TGF-β, IL-4, IL-10, and IL-1RA (Aggarwal and Pittenger, 2005; Uccelli et al., 2008; Murphy et al., 2013). This dynamic signaling promotes T-cell regulation and a shift to the anti-inflammatory M2 macrophage phenotype (Murphy et al., 2013; Pittenger et al., 2019). Furthermore, MSCs demonstrate antibacterial activity through a different mechanism, namely the production of LL-37, prostaglandin E_2_, and indoleamine 2,3-dioxygenase (Németh et al., 2009; Krasnodembskaya et al., 2010; Bonfield et al., 2013; Murphy et al., 2013). MSCs have also been shown to respond differentially to hypoxic conditions, mechanical stimulation, and even the physical structure of their environment (Kusuma et al., 2017), altogether demonstrating the profoundly dynamic nature of MSCs. Understanding this, it is reasonable to assume that biological changes in the body as a result of harmful environmental exposures may negatively affect patients’ responsiveness to MSC therapies. However, more research is needed to characterize the interaction between MSCs and the neural exposome.

## 7 Ethical concerns of MSC therapy

As mentioned previously, MSCs lack the ethical controversy inherent to embryonic stem cells because they can be harvested from adult tissue, thereby circumventing the need to procure stem cells from human embryos (Bacakova et al., 2018). Despite this major advantage, MSCs are not without ethical concerns.

Foremost among these concerns is the rise of unregulated stem cell clinics within the past decade. The promise of stem cell therapies as a category sparked demand, but slow development combined with lax regulatory environments in some countries, namely the United States, China, India, Mexico, and Thailand among others (Connolly et al., 2014), have given rise to many unregulated stem cell clinics (Lyons et al., 2022; Langford and Foong, 2024). This problem is not unique to MSCs, but MSCs are highly marketed and utilized by such clinics (Turner, 2021). These unregulated clinics offer stem cell treatments that are unproven at best and dangerous at worst. One particular case study covers a 74-year-old man who received intrathecal stem cells from a clinic in Russia for chronic fatigue. Following therapy, he developed lower extremity weakness and urinary retention secondary to abnormal lymphocytic and glial cell proliferation within the thoracolumbar thecal sac (Madhavan et al., 2020). Another such patient received unproven intrathecal stem cell injections in China, Argentina, and Mexico and subsequently developed low back pain, paraplegia and urinary incontinence secondary to a glioproliferative lesion of the thoracic spinal cord determined to be of non-host origin (Berkowitz et al., 2016).

The medical dangers of unproven stem cell treatments comprise only one aspect of the issue. Treatments received abroad can total anywhere between $10,000 and $60,000 per treatment according to patients having received such treatments (Petersen et al., 2014). In the U.S. few clinics advertise prices, but available price information ranges from $1,200 to $28,000 depending on the clinic and type of treatment (Turner, 2021). The United States reportedly has the highest number of unregulated clinics (Lyons et al., 2022), and this number has only continued to grow within the past decade. Estimates in 2021 reported that approximately 1,480 U.S. business operated around 2,754 clinics which offered unlicensed stem cell therapies (Turner, 2021). These clinics commonly claimed to treat pain, orthopedic conditions and injuries, and neurological diseases as well as provide aesthetic treatments for aging, hair loss, and other cosmetic indications (Turner, 2021). Additionally, these unregulated clinics engage in direct-to-consumer advertising (Turner and Knoepfler, 2016), typically through the internet and social media (Lyons et al., 2022), and often avoid branding themselves as stem cell or regenerative medicine clinics, choosing instead to market themselves as specialty pain, orthopedic, integrative medicine or other wellness clinics (Turner, 2021). Such advertising practices raise concerns about patients’ ability to give informed consent to receiving unproven treatments (Langford and Foong, 2024). It is clear is that many of these businesses do not comply with FDA regulations regarding the use of human cell products (Turner and Knoepfler, 2016; U.S. Food and Drug Administration, 2020), despite claims to the contrary (Turner, 2021). The continued existence of unregulated stem cell clinics and stem cell tourism as a practice could result in lasting damage to the clinical translation and public perception of stem cell therapies as a whole.

Another ethical and safety concern that must be addressed in the development MSC therapies is tumorigenicity. As mentioned previously, the risk of tumorigenicity of MSC products is present yet far lesser than the risk from ESCs or IPSCs (Ben-David and Benvenisty, 2011). Responsible preparation of MSC products must ensure that modifiable factors affecting risk of tumorigenicity are minimized. Of primary concern is the *ex vivo* expansion of MSCs, which may result in DNA damage due to reduced DNA polymerase efficiency and DNA repair mechanism dysfunction, thus leading to tumorigenesis and reduced functioning (Neri, 2019; Yamaguchi et al., 2024). Despite the potential risks, very few examples of spontaneous *in vitro* human MSC malignant transformation have been recorded (Pan et al., 2014). This evidence can be reconciled with previously discussed case reports of apparent tumor-like growths in patients having received stem cell therapies as these therapies were unproven and unregulated (Berkowitz et al., 2016; Madhavan et al., 2020). In the larger context of clinical trials, recent systematic reviews on the adverse effects of MSCs administered intravascularly in human clinical trials have revealed no increased risk of malignancy, along with other adverse effects, in patients who received MSCs compared to controls (Lalu et al., 2012; Thompson et al., 2020).

A final ethical consideration in the development of MSC therapies is the practice of biobanking of MSCs. Biobanking refers to the establishment of facilities that can store and maintain large number of stem cells from many donors, both for therapeutic and research purposes (Riegman et al., 2008). One immediate ethical concern concerning the use of stem cell biobanks is informed consent. Clear informed consent is crucial so that donors understand the implications of the present and future use of their cells, including the creation and potential immortalization of new cell lines derived from their tissues (Petrini, 2010; King and Perrin, 2014). Stem cells also contain an individual’s genetic information that is impossible to deidentify, which raises concerns about who has access to that information (Ashcroft and Macpherson, 2019). While biobanking improves access to MSC technologies, the potential of unequal access to biobanks is yet another ethical issue. When access is constrained by financial resources or geography this can perpetuate existing disparities in healthcare access. Stem cells retrieved from biobanks for clinical purposes must be HLA matched, and there is a relative scarcity of available specimens compatible with patients belonging to racial and ethnic minority groups (Shearer et al., 2017). There is also a lack of international standardization for biobanking of stem cells which complicates regulatory oversight (Shearer et al., 2017). This leads to heterogeneity of cell products and may affect genetic integrity (Neri, 2019), ultimately limiting viability for future research and therapeutic applications.

## 8 Challenges of MSC therapy

The most pressing challenge for MSC therapies is translating preclinical results to humans. Preclinical studies in rodent and canine models show tremendous promise in treating conditions of the CNS, and while phase 1 and 2 clinical trials in humans have proven the unambiguous safety of MSC treatments, they have collectively shown only a modest clinical benefit at best (McCulloch and Till, 2005). Several factors may explain the disappointing performance of MSCs in human clinical trials to date. The first is the quality of the models used to study these diseases. Animal models are not entirely representative of the complexity of neurologic disease in humans, especially in neurodegenerative conditions. Animal models of neurodegenerative diseases typically follow a familial disease paradigm, rather than the sporadic disease paradigm that is more commonly seen in humans (Götz et al., 2018). While the core pathological features of familial and sporadic neurodegenerative disease may be the same, there are notable differences in their etiologies (whether genetic environmental, or both) and their patterns of onset. Familial disease often has an earlier onset than sporadic disease (Piaceri et al., 2013; Franco et al., 2019), and may even be associated with different genetic risk factors than those typically contributing to sporadic disease (Nussbaum and Ellis, 2003). Clinical translation from preclinical to clinical trials in neurologic injury, such as stroke and TBI, may be limited in part due to the differences in biology and neural architecture between humans and commonly used rodent models (Vink, 2018). Therefore, it should not be surprising that a treatment tailored to a specific pathology or biological model does not translate effectively when applied to broader and/or more complicated biological system.

Another challenge is the survival rate of transplanted MSCs. Less than 1% of MSCs survive following transplantation (Pittenger et al., 2019). Culturing MSC under biological conditions (Wei et al., 2012) along with genetically engineering MSCs to overexpress certain growth factors (Horita et al., 2006; Jeong et al., 2014; Ghazavi et al., 2017) (as discussed previously) may increase survival and therefore increase regenerative effect. What’s more, the route of administration may affect the clinical efficacy of MSCs. Many clinical trials have used an intravenous approach, but there is evidence that MSCs administered intravenously get stuck in the lung vasculature (Harting et al., 2009; Masterson et al., 2021), though the clinical significance of this finding is unclear considering that MSCs administered intravenously have still shown benefit in some clinical trials (Cox et al., 2017; Levy et al., 2019; Cox et al., 2024). Ultimately, the best route of administration may be different for each disease (Pittenger et al., 2019).

Yet another hurdle in MSC translation from preclinical models to therapeutic use is the method of preparation and preservation of MSCs. The method of cryopreserving MSCs in the interim between harvesting and infusion has been thrown into question. MSCs used in animal models of disease are not usually frozen prior to infusion, whereas it is a common practice to cryopreserve the MSC products used in human clinical trials prior to infusion. This practice may result in MSCs that are less potent in their immunosuppressive effects (Moll et al., 2016; Bahsoun et al., 2019). There is also no standardization for cryopreservation procedure (Yong et al., 2015). The same can be said of the potency assays used to assess MSCs prior to clearance for administration. Commonly used *in vitro* potency assays include MSC activation assays, immune cell inhibition assays (usually T-cells and peripheral blood mononuclear cells), immune cell migration assays, regulatory T-cell induction assays, MSC secretome analysis, and quantitative RNA analysis of immune-related gene expression (Galipeau et al., 2016; Chinnadurai et al., 2018). However, there is no formal standardization of these methods, which ultimately results in significant heterogeneity in the development, handling, and characterization of the MSC product (Krampera et al., 2013; Galipeau et al., 2016; de Wolf et al., 2017; Torrents et al., 2023). There is an argument to be made that potency and release assays should be specific to the disease, cell-type, route of administration, and patient population (Pittenger et al., 2019), but even then, sub-criteria should still be standardized to ensure homogeneity of the product. Additionally, creation of good manufacturing practice (GMP) facilities for the manufacturing and scaling of MSC products presents logistical challenges (Dodson and Levine, 2015), not the mention that the practice of mass *ex vivo* expansion of MSCs in biotechnology facilities gives rise to senescent cells, which may also be less efficacious than non-senescent MSCs (Sensebé et al., 2013; Galipeau and Sensébé, 2018; Sugimoto et al., 2021). On the other hand, large-scale production of immortalized MSCs to generate exosomes may result in phenotypic changes that have yet to be fully explored (Chen et al., 2011). Finally, robust quality control measures are crucial for the clinical translation of MSC therapies. Consistency of the cell product is key to the consistency of the results, especially when manufacturers have considerable latitude regarding quality control measures and potency assays used to characterize their cells (Kolkundkar et al., 2014).

## 9 Conclusion

In this review we have discussed the history and development of the MSC, its characteristics, mechanisms of action, and clinical applications within the CNS. We review the current landscape of clinical trials surrounding MSCs for neurologic pathologies, focusing on notable landmark trials and promising upcoming studies while also addressing the relative paucity of phase 3 clinical trials for a number of CNS diseases. There is a long way to go and many hurdles to overcome before MSC therapies for CNS conditions gain regulatory approval, but as the field of research grows, exciting advances continue to be made. Among these are MSC-derived exosome therapies independent of the cell itself, creation of MSC lines that produce trophic factors, and co-implantation with biological scaffolds. Alongside these advances, research continues on the optimal sourcing, culturing, preparation, dosage, and administration strategy for each specific MSC-line and condition. Altogether, MSCs represent a promising therapeutic strategy for treating injury and disease of the CNS. Though FDA approval is yet to be granted to any MSC therapies for the purpose of treating CNS conditions, the recent FDA approval of a MSC therapy (Ryoncil) for treatment of pediatric steroid-refractory graft-vs-host disease (Kurtzberg et al., 2020; Pflaum, 2024) demonstrates the clinical need for these drugs and paves the way for future approvals of MSC therapies.
